# A Systematic Review and Lived-Experience Panel Analysis of Hopefulness in Youth Depression Treatment

**DOI:** 10.1007/s40894-021-00167-0

**Published:** 2021-07-06

**Authors:** Clio Berry, Joanne Hodgekins, Daniel Michelson, Laura Chapman, Olga Chelidoni, Lucie Crowter, Catarina Sacadura, David Fowler

**Affiliations:** 1 Brighton and Sussex Medical School, University of Sussex, Brighton, UK; 2 Norwich Medical School, University of East Anglia, Norwich, UK; 3 School of Psychology, University of Sussex, Brighton, UK; 4 School of Life Sciences, University of Sussex, Brighton, UK; 5 Research & Development, Sussex Partnership NHS Foundation Trust, Worthing, UK; 6 Primary Care and Public Health, Brighton and Sussex Medical School, University of Brighton, Watson Building, Falmer, BN1 9PH UK

**Keywords:** Hopefulness, Depression, Adolescence, Youth mental health, Psychological therapy

## Abstract

Hopefulness is arguably of central importance to the recovery of youth with major or complex youth depression, yet it is unclear how hopefulness can best be enhanced in treatment. A narrative synthesis of published and grey literature was combined with new insights from a youth lived-experience panel (N = 15), focusing on to what extent and how specific psychological therapies and standard mental health care scaffold hopefulness as applied to depression among 14–25-year-olds. Thirty-one studies of variable quality were included in this review; thirteen were qualitative, thirteen quantitative, and five used mixed methods. Hopefulness is an important active ingredient of psychotherapies and standard mental health care in youth depression. Evidence suggests talking and activity therapies have moderate to large effects on hopefulness and that hopefulness can be enhanced in standard mental health care. However, varying intervention effects suggest a marked degree of uncertainty. Hopefulness is best scaffolded by a positive relational environment in which there is support for identifying and pursuing personally valued goals and engaging in meaningful activity.

Animated (https://www.youtube.com/watch?v=o4690PdTGec) and graphical summaries (https://doi.org/10.13140/RG.2.2.27024.84487) are available.

**Supplementary Information:**

The online version contains supplementary material available at 10.1007/s40894-021-00167-0.

## Introduction

Hopefulness, a long-standing object of philosophical and religious interest (Snyder, 2000), was recognized as central to psychoanalysis (Freud, 1953) and a basic—albeit elusive—ingredient in psychiatry (Menninger, 1959). Yet hopefulness gained little further attention until the 1990s, with the creation of the cognitive model (Snyder, 2000) and the identification of hope as a core instigator and process variable within the mental health recovery movement (Schrank et al., 2008). Many have argued hopefulness underlies potentially all psychotherapeutic change (Gallagher et al., 2020; Taylor, 2000); yet this assumption is unmatched by research activity (Koehn & Cutcliffe, 2007). Evidence of the role of hopefulness within mental health interventions remains limited, especially in youth (Gallagher et al., 2020). Hopefulness may be especially important in adolescence (Berry & Greenwood, 2017); a key time for developing sense of self and future aspirations (Oyserman, 2001) but also of mental health vulnerability (Kessler et al., 2007). Existing interventions in youth depression, both specific psychotherapies and general standard mental health care, seemingly have only modest effects (Eckshtain et al., 2020); producing unreliable or no symptom improvement for at least 50% of youth (Bear et al., 2019) and often not improving social recovery, especially for youth with marked social and occupational withdrawal (Fowler et al., 2010). Such withdrawal is not only personally and economically important, but additionally predicts worsening symptoms (Goldman-Mellor et al., 2016). Therefore, increasing understandings of the interventional role of hopefulness has the potential to improve youth treatment and outcomes. This study provides an inclusive review of current evidence on the role of hopefulness in the treatment of youth depression, and combines this evidence with new insights generated by a youth panel with lived experience of mental health problems.

The cognitive model defined hopefulness as goal-directed cognition, comprising self-agency (motivation and belief in one’s ability to progress towards goals) and pathways (identification of specific means of goal pursuit) (Snyder, 2000). Unlike more emotion and faith-based models (Clarke, 2003; Herth, 1991), cognitive hopefulness is especially amenable to intervention and is less confounded with symptomatology or spiritual beliefs. Hopefulness is distinct from alternative positive self and future construals, such as self-efficacy and optimism (Alarcon et al., 2013), for it predicts unique variance in health and wellbeing (Magaletta & Oliver, 1999) and is more separable from personality traits (Alarcon et al., 2013). Hopefulness is additionally neurologically distinct from hopelessness and the two can co-exist (Jevne, 2005; Nunn, 1996). Hopefulness robustly predicts psychological, social and occupational wellbeing and reduced mental health symptoms for students (Griggs, 2017), adolescents (Esteves et al., 2013), and adolescents with chronic illnesses (Griggs & Walker, 2016), with seemingly more predictive validity than negative self-beliefs (Berry & Greenwood, 2017). Hopefulness is positively future-oriented and resilience-building. It protects against the impacts of adversity (Valle et al., 2006), and the negative prospective cognition (Bjärehed et al., 2010), difficulty in vividly imagining (Morina et al., 2011) and expecting positive future events (Thimm et al., 2013), and suicidality (Hirsch et al., 2012) characteristic of depression. Depression with anxiety is as common as without (Kessler et al., 2015), and comorbidity is linked to greater severity (Costello et al., 1996) and reduced recovery (Edbrooke-Childs et al., 2018). Hopefulness is therefore even more important in diagnostic complexity (Fowler et al., 2019), with such complexity especially common in youth (Blazer et al., 1994).

The increased use of positively-oriented hopefulness-focused treatment may better enact therapeutic change through augmenting information-processing (Nelson et al., 2009) and engendering positive emotions (Schubert et al., 2020). Hopefulness can be a central feature of specific psychotherapies, for example in Hope Therapy, a form of Cognitive Behavioral Therapy (CBT) based on cognitive hope theory (Snyder, 2000). This intervention has some evidence of effectiveness, but studies in adolescence and clinical populations are very limited (Weis & Ash, 2009). More broadly, hopefulness is arguably a feature of all psychotherapy; for successful treatment depends on the collaborative identification and pursuit of goals, and increased self-agency and pathways-thinking, for example through understanding how different therapeutic techniques can facilitate desirable outcomes (Weis & Ash, 2009). Hopefulness is equally as relevant to standard mental health care, for example within the therapeutic relationship between patients and professionals (Berry & Greenwood, 2015), and is linked to positive outcomes in this setting (Schrank et al., 2012). Yet, better understandings are needed as to how hopefulness is engendered in treatment and how it impacts on symptomatic and social recovery outcomes.

## Current Study

Hopefulness is a promising candidate to improve both specific psychotherapies and standard mental health treatment of youth depression, yet better understandings are needed of the impacts of hopefulness in these different settings. The current systematic review aimed to generate a comprehensive synthesis of research evidence pertaining to the therapeutic enhancement and outcomes of hopefulness for youth with depression, first asking what is the evidence that hopefulness in both specific psychological therapies and standard mental health care leads to improvements in depression and social recovery for youth with major or complex depression (Research Question 1)? In addition to the need to consider different treatment settings, the context within which youth live and the diversity of their life experiences may influence the degree to which they feel hopeful (Hughes et al., 2010). Therefore, further study is needed to explore in what settings and contexts, and for whom, hopefulness is most important or effective (Research Question 2). Moreover, whilst evidence suggests hopefulness may impact on outcomes through augmenting information-processing and affect, questions remain as to what are the specific processes through which hopefulness arises and impacts on symptomatic and social recovery outcomes in youth depression treatment (Research Question 3)? The current objective was to create an inclusive synthesis, involving both published and grey literature evidence, and including research using any quantitative, qualitative or mixed methodology. In addition, lived experience participation was included as a component in the evidence synthesis in order to combine rigorous systematic review methods with experiential knowledge (Harris et al., 2016) in answering the research questions.

## Methods

### Protocol and Registration

This review was registered on PROSPERO on 14/07/2020 (CRD42020192701).

### Study Search

The academic databases ASSIA, CINAHL plus, PsychArticles, PsychInfo, PubMed/Medline, Scopus, and Web of science were searched between the 18th and 19th June 2020 using terms reflecting the age range, hopefulness, depression, psychotherapeutic or mental health treatment setting, and research design. Full search terms are provided in appendices (Online Appendix A). Open access thesis (EThOS, OATD, EBSCO) and grey literature (OpenGrey) depositories, and youth and student mental health organization websites (including YMCA, Student Minds, Anna Freud Centre), were searched between 1st July and 7th August 2020. Reference lists of 15 existing reviews of hopefulness for youth and/or clinical populations (see Online Appendix A) and of all included studies were screened. Screening and selection were managed using Covidence software (Veritas Health Innovation, 2020).

### Study Selection

Inclusion criteria were that studies were interventional or observational, used qualitative, quantitative or mixed methods, evaluated a specific psychological intervention or standard mental health care, and had a majority sample aged 14–25 years and meeting depression caseness, irrespective of comorbidity. The upper age limit of 25 years was selected to match typical youth and youth mental health service coverage. Qualitative studies reporting a clinical sample with diagnoses explicitly described as including depression were included. Studies in any health, community, or educational setting in any geographical locality were included. Studies in non-English language, which were non-peer reviewed (not including grey literature additions) or which presented no primary data were excluded. Full criteria are provided in Online Appendix B. Study screening was conducted by five reviewers. Disagreements between reviewers was managed through all reviewers discussing the full text of each record and making a consensus decision on inclusion or exclusion, or taking the majority decision in the absence of full consensus.

### Data Extraction

Data were extracted using Covidence and Excel. Extracted data included sample characteristics, design, methods, analysis, intervention characteristics (interventionist, setting, content, sessions, mode and delivery), quantitative data (absolute measure scores, standard deviations, frequencies, within and/or between group effect sizes at pre-post-intervention and follow-up as available), and qualitative data (higher order and subthemes). Intervention outcomes of interest were diagnostic and symptomatic changes in depression, captured using any diagnostic interview or other observer or self-report scale, and changes in social recovery. Social recovery can be understood as (re)gaining functioning with respect to time spent in valued and meaningful social and occupational activities (Hodgekins et al., 2015). Data extraction was performed by five reviewers.

### Youth Lived Experience Panel

Fifteen youth aged 15–24 years of different genders (53% female) and nationalities formed a lived experience consultation panel. The panel had experience of low mood or depression and many had experience of mental health treatment. The panel was recruited from a mental health NHS Trust, youth mental health and community services, and national youth and student mental health networks. Involvement was reimbursed. In two virtual Zoom (Zoom Video Communications Inc., 2020) 2-h meetings and/or email participation, the panel used self-selected images or objects to discuss their concepts and experiences of hopefulness. The panel were not involved in the process of setting the review questions or in delivering the review methods. The panel were involved in the interpretation of the review findings. Moreover, the panel were asked for their own answers to the research questions in order to provide insights perhaps not captured in research evidence reviewed. In addition, nominal group process methods (McMillan et al., 2016) were used in a research priority setting exercise. First the panel freely and independently generated future priorities for research on hopefulness for youth with major or complex depression, creating an online “idea bank” using MURAL software (Tactivos Inc DBA MURAL, 2020). After being presented with a detailed summary of the emerging review findings, the panel generated additional research priorities and then independently voted for their top 10 priority ideas. Finally, in two subgroups, the panel was asked to rank the top 10 priorities from one to 10 using imagined financial research investment from £5 million to £500,000. The two subgroups were asked to share their respective rankings and reach an overall consensus. The group were unable to reach consensus, and instead were invited to independently rank using a ranked choice question presented via Qualtrics software (Qualtrics, 2020) after the final panel meeting.

### Risk of Bias

Risk of bias within each study was rated with the Mixed Methods Appraisal Tool (MMAT) (Hong et al., 2018, 2019), using the two filter questions and then five qualitative, quantitative (RCT, non-randomized or descriptive), or mixed methods criteria as appropriate. A GRADE assessment (Oxman, 2004) of bias risk across all studies was generated.

### Synthesis of Results

A narrative evidence synthesis (Popay et al., 2006) was produced, synthesizing research evidence and lived experience insights. The PRISMA statement (Moher et al., 2009) was used to prepare this report. Scientific and lived experience evidence was synthesized in relation to the research questions. Within and/or between-group effect sizes were narratively described and summarized. Qualitative data were additionally narratively described and summarized. Insights from the lived experience panel were narratively described with respect to their relevance for each of the three research questions. Verbatim quotes from lived experience experts are provided in italics.

## Results

### Study Selection

**Fig. 1:**
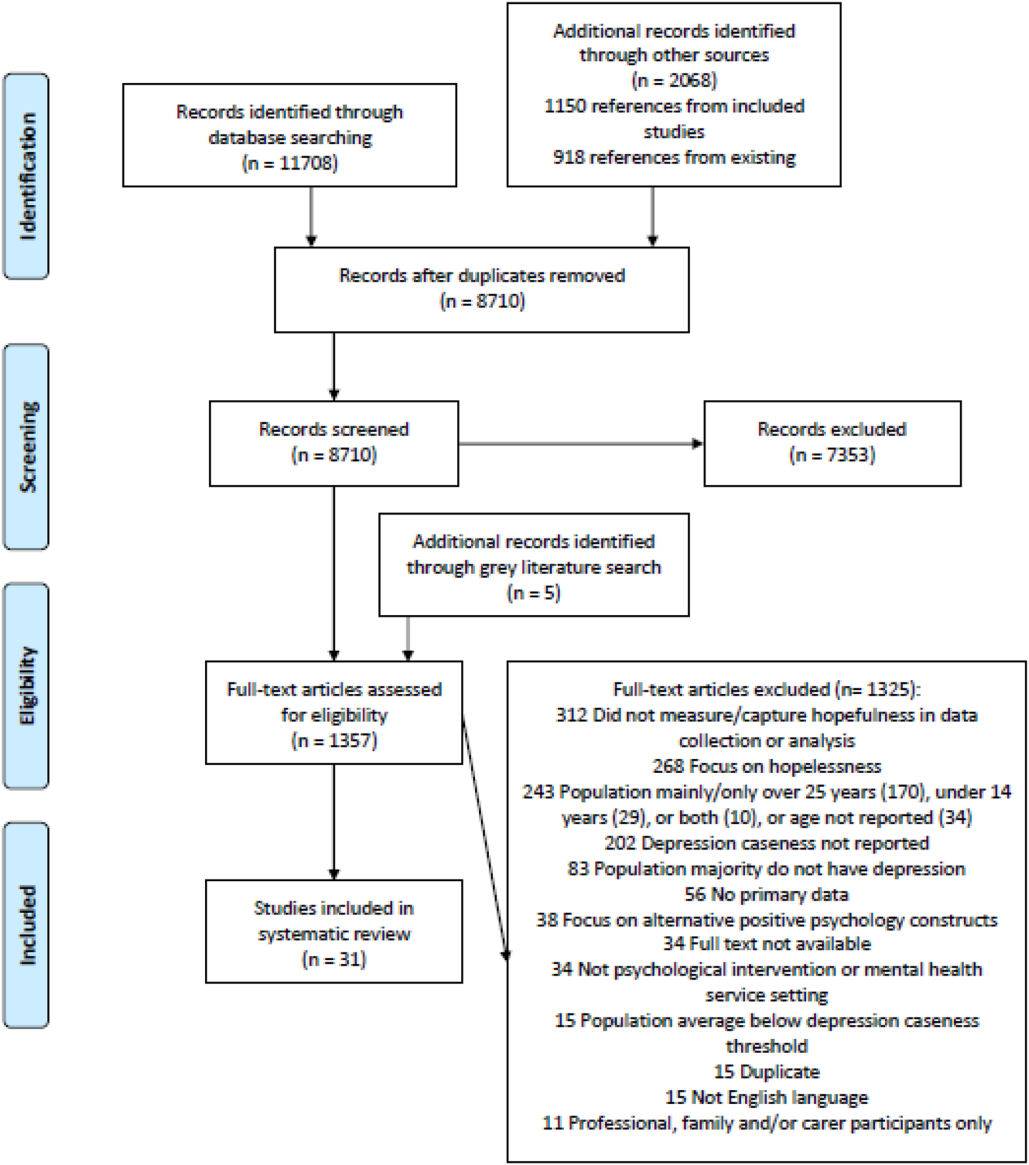
PRISMA diagram (Moher et al., 2009) of study selection

Five reviewers screened 8710 records (see Fig. 1) using the title and abstract. The first author screened all studies with four other reviewers independently screening 3036 records (34.87%). Reviewer agreement regarding whether each record met inclusion or exclusion criteria was 83.64%. At the full text stage, all records were screened by the first author and one of the four other reviewers independently, with reviewer agreement at 96.20%.

### Study Characteristics and Conceptualization of Hopefulness

Thirty-one studies were included in this review (Table 1). Thirteen studies were qualitative, 13 quantitative, and five used mixed methods. Five studies employing quantitative methods were Randomized Controlled Trials (RCT), ten were non-randomized or uncontrolled pre-post or follow-up studies. Six qualitative and mixed methods studies were adjunctive or sub-studies to RCTs. Nine studies were conducted in the US or Canada, six in Europe, five in the UK, and five in Australia or New Zealand. Three studies were theses (Conklin, 2009; Davidson, 2008; Hambridge, 2017); all others were published journal articles.

**Table 1:** Study characteristics

First author	Date	Aim	Design	Setting, Country	Sample (N)	Age years M(SD), range	Gender n(%)	Ethnicity n(%)	Depression caseness
Anttila	2014	Describe adolescents’ concerns and hopes at referral to adolescent psychiatric outpatient treatment	Cross-sectional qualitative RCT sub-study	Outpatient adolescent mental health services, Finland	Adolescents involved in Depis.net trial for major depression (70)	-(-), 15–17	Female 54(77), Male 16(23)	-(-)	RCT participants referred by adolescent psychiatric outpatient services
Aubuchon-Endsley	2014	Examine psychometric properties of Milwaukee Psychotherapy Expectancies Questionnaire	Quantitative cross-sectional within and between-groups scale validation	University psychology clinic, US	Students accessing university counselling center (55)	27.0(-) [Mode = 21], 18–58,	Female 29(52)	-(-)	BDI-II mean score = 22.21
Binder	2013	Explore adolescents’ experiences of taking part in psychotherapy	Cross-sectional qualitative	Outpatient adolescent mental health services, Norway	Youth accessing two child and adolescent outpatient clinics (14)	-(-), 16–18	Female 8(66), Male 6(34)	Norwegian 12(86)	Clinical sample with problems including depression
Bury	2007	Develop in-depth understanding of youth experiences of individual psychoanalytic psychotherapy, including referral, engagement, therapist, therapeutic relationship, most and least helpful factors	Cross-sectional qualitative	Community mental health clinic, UK	Youth attending at least 6 months of psychotherapy, ceasing at least 3 months prior (6)	-(-), 17–21	Female 4(67), Male 2(33)	-(-)	Clinical sample with problems including depression
Conklin	2009	Assess whether two-session goal intervention improves goal progress, depression, state hope, and state anxiety	Quantitative RCT	University, US	Undergraduate psychology students (103)	Intervention 20.3(5.1), Control 20.4(6.4)	Female 57(55)	White 81(79), Asian-American 9(9), other 6(6), Hispanic/Latino 4(4), African-American 3(3)	BDI-II mean score intervention = 16.5, control = 18.8
Davidson	2012	Examine adolescents’ thoughts on therapy; specifically, therapeutic relationship and its role in personal disclosure-related comfort	Cross-sectional qualitative	Child and youth mental health center, Canada	Youth engaged in psychotherapy in child and youth mental health office (15)	16.0(1.96), 13–19	Female 10 (67), Male 5 (33)	-(-)	Clinical sample with diagnoses including depression
Dowling	2015	Explore progress and depth of counselling processes used in online chat and associations with session attendance and outcomes	Quantitative uncontrolled pre-post within-group	Online counselling service, Australia	Youth engaged in online counselling with youth mental health service (49)	-(-), 16–25 Median = 17	Female -(86)	-(-)	Clinical sample with depression (65%)
Fowler	2018	Assess effectiveness of early intervention in psychosis (EIP) services augmented with Social Recovery Therapy for patients with social disability in the context of first episode psychosis	Quantitative RCT	NHS community EIP services, UK	Youth with non-affective psychosis experiencing social disability after 12–30 months engagement in EIP services (154)	24.4(-), 20–29	Male 116(75), Female 38(25)	White 113(73), Asian/White and Asian 18(12), Black/White and Black African or Caribbean 11(7), Mixed and other 13(8)	BDI-II mean score intervention = 18, control = 19
Gabrielsen	2019	Assess effectiveness of wilderness therapy program in improving self-efficacy, depression, anxiety, life satisfaction, physical health, distress and self-coherence	Mixed methods^a^, uncontrolled pre, post and follow-up within-group	Public mental health care, Norway	Youth referred to mental health care system (32)	16.5(0.6), 16–18	Female 21(65), Male 11(35)	-(-)	HADS mean score = 8.5
Gee	2016	Assess participants’ experiences of Social Recovery Therapy and treatment as usual	Cross-sectional qualitative RCT sub study	NHS secondary mental health care and non-NHS youth services, UK	Youth engaged in PRODIGY trial for social disability and severe and complex mental health problems (17)	-(-), 16–25	Female 9(53), Male 8(47)	-(-)	Research diagnosis of depression or dysthymia (53%)
Gillig	2019	Assess effectiveness of camp program in improving wellbeing for youth who identify as LGBTQ	Mixed methods, uncontrolled pre-post within-group	Summer camp, US	Young campers who identify as LGBTQ (56)	15.4(1.8), 12–20	Female 17(31), Gender non-conforming 15(27), Male 12(21), Transgender male 8(14), Transgender female 2(3), Unsure/Questioning 2(3)	White –(77), Latino –(16), Other –(7)	CES-D-4 mean score = 2.7
Green	2007	Examine efficacy of cognitive behavioral solution-focused life coaching program in enhancing cognitive hardiness and hopefulness	Quantitative RCT	High school, Australia	Female high school students (56)	16.1(-), 16–17	Female 56(100)	-(-)	DASS-21 depression mean score = 12.1
Hambridge	2017	Explore experiences of and impacts of care farm engagement	Mixed methods, non-randomized controlled pre, post, and follow-up within and between-groups	Care farm alternative to mainstream education, UK	Youth with behavioral, social, emotional and school functioning problems, 98% with neurobehavioral or learning disability (50)	Intervention 14.4(0.9), Control 13.1(0.4)	Male 36(72), Female 14(28)	Not stated	DASS-21 depression mean score intervention group = 11.12, control = 6.24
Isa	2018	Assess effects of psychological intervention that includes psychoeducation and basic elements of cognitive behavioral therapy	Quantitative uncontrolled pre-post within-group	Child and adolescent psychiatric outpatient clinic, Nigeria	Youth with depression attending psychiatric clinic, taking antidepressant medication for at least 3 months (18)	15.5(1.5), 13–18	Female 9(50), Male 9(50)	-(-)	BDI mean score = 24.44
Leavey	2005	Explore and describe phenomenon of the process of becoming, living with, and recovering from mental health problems as experienced by transition-aged youth	Cross-sectional qualitative	Community psychosocial rehabilitation center, Canada	Youth attending psychosocial rehabilitation center (13)	-(-), 17–23	Male 7(54), Female 6(46)	-(-)	Clinical sample with diagnoses including depression
Leibovich	2020	Examine what allows psychotherapeutic interpretation to facilitate growth and promote flourishing	Mixed methods case study	Pilot case of psychotherapy RCT, Israel	Individual pilot psychotherapy client who self-referred to study (1)	-(-), 21	Female 1(100)	-	Met Major Depressive Disorder diagnostic criteria, BDI score = 22
Lin	2013	Assess effectiveness of forgiveness intervention	Quantitative RCT	University counselling centers, Taiwan	University students with high depression and anxiety, low forgiveness and insecure maternal attachment (27)	-(-), 18–23	-(-)	-(-)	CES-D mean score intervention group = 23.00, control group = 25.25
Lin	2014	Assess effectiveness of grief-processing-based psychological group intervention in improving trauma, distress, and hopefulness	Quantitative non-randomized controlled pre-post within and between-groups	Government-funded orphanages, China	Youth whose parents had died from HIV/AIDS (124)	13.6(-), 9–17	Male 76(61), Female 48(39)	Chinese Han 124(100)	CES-DC mean score = 39.00
Metsӓranta	2019	Explore use and effectiveness of online e-diary intervention for adolescent depression	Mixed methods longitudinal RCT sub study	Adolescent psychiatry outpatient clinics, Finland	Participants of the Depis.net trial for depression (89)	16(-), 15–18	Female 66(75), Male 22(25)	-(-)	BDI score above 16 (58%)
Midgley	2016	Explore hopes and expectations for therapy among adolescents with depression	Qualitative RCT sub study	Child and Adolescent Mental Health Services, UK	Participants of the IMPACT trial for depression (77)	15.9(1.77), 11–17	Female 55(77), Male 22(23)	-(-)	K-SADS diagnosis of moderate to severe depression
Pingitore	2017	Explore adolescents’ perceived benefits, meaningfulness and experiences of participating in process-oriented group psychotherapy and recommendations for future providers	Cross-sectional qualitative	Children’s hospital outpatient group psychotherapy services, US	Adolescents in active therapy and medical treatment, engaged in psychotherapy group for 3 months (10)	15.8(-), 13–18	Female 10(100)	Caucasian 9(90), Hispanic 1(10)	Clinical sample with diagnoses including depression, all with prior inpatient admission/s
Rayner	2018	Develop thematic model of youth recovery	Cross-sectional qualitative	Community youth mental health support services, Australia	Youth accessing community youth mental health support services (15)	20(-), 18–23	Females 10(67), Male 5(33)	-(-)	Clinical sample with problems including depression
Ritschel	2011	Evaluate effectiveness of adapted behavioral activation manual for reducing depression among adolescents	Quantitative uncontrolled pre-post within-group pilot	Outpatient adolescent mood clinic, US	Youth accessing outpatient adolescent mood clinic or self-referring to study (6)	15(1), 14–17	Female 3 (50), Male 3 (50)	Caucasian 3(50) Biracial1(16.6), African-American 2(33.3)	K-SADS diagnosis of depression and CDRS-R score of at least 45
Ritschel	2016	Further evaluate feasibility and potential efficacy of adapted behavioral activation manual for improving depression and psychological wellbeing	Quantitative uncontrolled pre-post within-group pilot	Outpatient treatment clinic, US	Youth accessing outpatient adolescent mood clinic or self-referring to study (28)	15.4(1.16), 14–17	Female 19(68), Male 9(32)	African American 11(39.3), Caucasian 10(35.7), Biracial 5(17.9), Asian 1(3.6), Hispanic 1(3.6)	K-SADS diagnosis of depression and CDRS-R score of at least 45
Sælid	2017	Test effectiveness of brief rational emotive behavior intervention against an attentional placebo and a control group in improving depression, anxiety, self-esteem, hopefulness and dysfunctional thinking	Quantitative RCT	High school, Norway	High school students with mild depression (62)	-(-), 16–19	-(-)	-(-)	HADS mean score = 12.47
Shepherd	2018	Explore Māori adolescents’ opinions about an online intervention for depression	Cross-sectional qualitative RCT sub-study	High school, New Zealand	Participants of the SPARX trial for depression (6)	14.7(-), 14–16	Female 5(84), Male 1(16)	Māori/taitamariki 6(100)	PHQ-9 score of 10–19
Smith	2011	Assess effectiveness of integrated yoga and meditation program in improving psychological, spiritual and physical wellbeing compared to a standard yoga program and inactive control	Quantitative non-randomized controlled pre-post within and between-groups	University, US	Students with at least mild depression, anxiety or stress (69)	21.2(4.2)	Female –(51), Male –(49)	White –(57), Other –(43)	DASS-21 depression mean score intervention group = 12.79, yoga-as-exercise group = 10.71, control = 5.87
Teodorczuk	2019	Assess effectiveness of positive psychology intervention in improving depression, mental health and hopefulness	Quantitative non-randomized controlled pre-post within and between-groups	Child and youth care center, South Africa	Adolescents in a residential youth care facility (29)	16.3(1.4), 14–18	Female 17(59), Male 12(41)	African 18(62)	RCADS-SV mean score = 58
Walsh	1997	Explore reactions of inpatient adolescents experiencing suicidality to an art future image intervention	Longitudinal qualitative interventional	Adolescent psychiatric inpatient unit, US	Inpatients of a unit for adolescents experiencing depression and suicidality (11)	15(-), 13–18	Female 7(84), Male 4(16)	-(-)	Clinical sample with problems including depression (100%)
Watsford	2013	Explore expectations of 12–25-year-olds regarding role as a mental health service “client”, therapist’s role, and expectations of therapy processes and outcomes	Cross-sectional qualitative	Youth mental health service, Australia	Youth presenting to youth mental health services for the first time (20)	17.3(12.6), 12–24	Female 11(55), Male 9 (45)	-(-)	Clinical sample with problems including depression
Weitkamp	2017	Investigate how adolescents with depression referred for psychodynamic psychotherapy experience their difficulties and their therapy expectations and hopes	Cross-sectional qualitative	Private and community outpatient psychotherapy clinics, Germany	Youth with depression entering psychotherapy with a maximum of two sessions attended (6)	-(-), 15–19	Female 5(83)	-(-)	K-SADS diagnosis of mild to moderate depression

^a^Only quantitative findings were extracted as the qualitative data was provided by young people’s parents. *BDI-II* Beck Depression Inventory II (Beck et al., 1996), using a clinical threshold of 13 (community sample) or 19 (psychiatric sample) (von Glischinski et al., 2019) for the depression caseness inclusion criterion in the present review, *HADS* Hospital Anxiety and Depression Scale (Zigmond & Snaith, 1983), using a clinical threshold of 8 (Bjelland et al., 2002), *CES-D* Center for Epidemiologic Studies Depression Scale (Radloff, 1977), using a clinical threshold of 16 (McDowell, 2006), *CES-D-4* 4-item CES-D (Melchior et al., 1993), using a clinical threshold of 3 (Zauszniewski & Graham, 2009), *DASS-21 depression* Depression, Anxiety and Stress Scales 21-item (depression subscale) (Lovibond & Lovibond, 1995), using a clinical threshold of raw score 5 (Gomez, 2016; Henry & Crawford, 2005; Reilly et al., 2018), *BDI* Beck Depression Inventory(Beck et al., 1961), using clinical thresholds for BDI-II, *CES-DC* Center for Epidemiologic Studies Depression Scale for Children (Fendrich et al., 1990), using a clinical threshold of 16 (Faulstich et al., 2017), *K-SADS* Schedule for Affective Disorders and Schizophrenia for School-Age Children (Ambrosini, 2000), *CDRS-R* Children's Depression Rating Scale-Revised, using a clinical threshold of 40 (Poznanski et al., 1984), *PHQ-9* Patient Health Questionnaire 9-item, using a clinical threshold of 10 (Kroenke et al., 2001), *RCADS-SV* Revised Child Anxiety and Depression Scale-Short Version (Ebesutani et al., 2012), using a clinical threshold of 21 (Lydon-Staley et al., 2019)

All studies measuring hopefulness specified it as an outcome and, in one study, an outcome (state hope) and outcome moderator (trait hope) (Conklin, 2009). Most studies used cognitive hopefulness measures and all but two studies focused on trait-level hope (Conklin, 2009; Gillig et al., 2019). All hopefulness measures used were self-report questionnaires (Online Appendix C). The lived experience panel deemed hopefulness to be both centrally important, *“Hope has been really key for my mental health for a very long time*”, and incredibly powerful; *“…a momentary moment of hope could lead to someone implementing massive change”*. The panel were asked to reflect on their concepts of hopefulness and then the fit of these with the cognitive model. The panel largely endorsed the cognitive model, stating that its goal-directed focus “*definitely resonates*” and emphasizing the importance and mutually reinforcing nature of self-agency and pathways thinking:

“…hope begins with having a goal and seeing a way for it to happen out of no way.”

“Not hope alone, but hope leading to action, that's really important.”

“There are ways around things, different pathways, I really relate to that.”

“It’s hard to have the knowledge of the pathways without having the goal and drive, both pathways and the desire work together.”

“I have a lot of hopefulness in life around several different areas and that this is made up of me knowing that I have the motivation to achieve things and the means (pathways) to achieve these things…If I don’t have any goals to pursue that I become less hopeful”

### Risk of Bias Within Studies

**Fig. 2:**
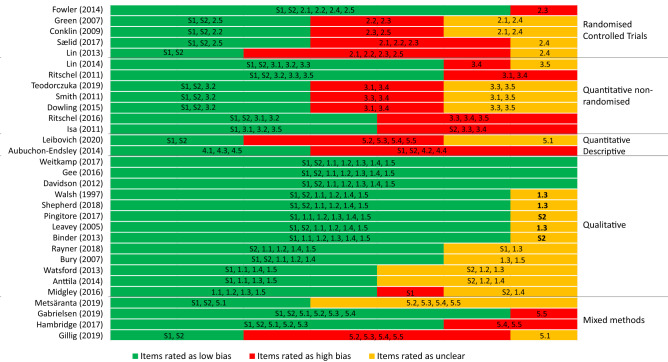
Risk of bias within individual studies. *Notes:* See supplementary figure note (Online Appendix D) for corresponding appraisal checklist items. For the purposes of quality appraisal, quantitative descriptive items were used for Leibovich et al. (2020). Whilst the case study is essentially mixed methods, for it presents both numerical and qualitative data with some integration, it is a case study and qualitative data were presented to illustrate quantitative ratings rather than providing any qualitative analysis. It was noted in addition that participation in the art future image intervention in the study conducted on an adolescent psychiatric inpatient unit (Walsh & Minor-Schork, 1997), could not be considered completely voluntary and this may have influenced participants’ responses

Studies were of variable quality, with 28.57–100% of elements rated as low bias (Fig. 2). Qualitative studies tended to be higher quality than quantitative and mixed methods studies, reflecting the high usage of non-randomized and uncontrolled quantitative designs. The items most commonly rated as high risk of bias were accounting for confounders in design and analysis and outcome data completeness (Fig. 2). The items most commonly rated as unclear were adequate data collected to answer the research questions, assessor blinding, and adequate derivation of qualitative findings from the data (Fig. 2). Studies rated as lower quality (Aubuchon-Endsley & Callahan, 2014; Gillig et al., 2019; Isa et al., 2018; Lin et al., 2013; Sælid & Nordahl, 2017; Smith et al., 2011; Teodorczuk et al., 2019) did not appear markedly different in reported interventional effects on hopefulness but reported larger effects on depression. Qualitative studies of lower quality (Anttila et al., 2015; Midgley et al., 2016; Watsford et al., 2013) were those which largely focused on hopes for therapy.

### Synthesis of Results

#### What is the Evidence that Hopefulness in Both Specific Psychological Therapies and Standard Mental Health Care Leads to Improvements in Depression and Social Recovery for Youth with Major or Complex Depression (Research Question 1)?

##### Specific Psychological Therapies

The specific psychological interventions identified were incredibly variable (see Online Appendix E), ranging from cognitive and/or behavioral-based therapies (Fowler et al., 2018; Gee et al., 2018; Isa et al., 2018; Lin et al., 2013, 2014; Metsäranta et al., 2019; Ritschel et al., 2011, 2016; Sælid & Nordahl, 2017; Shepherd et al., 2018) to other talking (Conklin, 2009; Green et al., 2007; Leibovich et al., 2020; Teodorczuk et al., 2019), arts (Walsh & Minor-Schork, 1997) or activity-based (Gabrielsen et al., 2019; Gillig et al., 2019; Hambridge, 2017; Smith et al., 2011) interventions. Interventions were variable with respect to duration and number of sessions (Online Appendix E). Most interventions were provided in mental health service settings—of which all but one (Walsh & Minor-Schork, 1997) were outpatient—or an educational setting, with two provided in residential care and two in a community nature-based setting (Table 1). Most studies involved community samples (Conklin, 2009; Gillig et al., 2019; Green et al., 2007; Lin et al., 2013, 2014; Sælid & Nordahl, 2017; Shepherd et al., 2018; Smith et al., 2011; Teodorczuk et al., 2019), others involved clinical (Fowler et al., 2018; Gabrielsen et al., 2019; Isa et al., 2018; Metsäranta et al., 2019) or mixed populations (Gee et al., 2018; Ritschel et al., 2011, 2016). There was an equivalent mixture of individual and group interventions (Online Appendix E). All but one (Shepherd et al., 2018) intervention were provided in person, mostly by mental health professionals.

Nearly all randomized or controlled studies reported significant between-group effects with respect to significantly increasing hopefulness and reducing depression pre- and post-intervention (Fowler et al., 2018; Green et al., 2007; Sælid & Nordahl, 2017; Smith et al., 2011), showing mainly moderate to large effects of Social Recovery Therapy (SRT), life coaching, and yoga and meditation (not yoga as exercise (Smith et al., 2011)) on these outcomes. Similarly, most uncontrolled studies reported significant within-group effects in increasing hopefulness and reducing depression (Gillig et al., 2019; Isa et al., 2018; Ritschel et al., 2011), showing mainly moderate to large effects of behavioral activation, psychoeducation and CBT, and an LGBTQ camping intervention on these outcomes. Only a positive psychology group intervention, versus a waitlist control, produced no between or within-group effects on hopefulness or depression (Teodorczuk et al., 2019). All other studies reporting no between-group differences (Conklin, 2009; Lin et al., 2013, 2014; Sælid & Nordahl, 2017; Teodorczuk et al., 2019) showed within-group increases in hopefulness in both interventions and comparators; the latter including attentional placebo (Sælid & Nordahl, 2017), goal-visualization (Conklin, 2009), social communication and perspective-taking (Lin et al., 2013), and an inactive control (Lin et al., 2014). Notably, effect sizes for hopefulness appeared greater for Rational Emotive Behavior Therapy versus its attentional placebo (Sælid & Nordahl, 2017) and for the grief-based intervention versus its inactive control (Lin et al., 2014). Two studies reported significant improvements in depression for interventions and active controls, with no evident between-group differences (Lin et al., 2013; Sælid & Nordahl, 2017). Studies which did not measure hopefulness (Gabrielsen et al., 2019; Hambridge, 2017; Shepherd et al., 2018; Walsh & Minor-Schork, 1997) provided qualitative evidence (Online Appendix F) that it was enhanced by the intervention.

Few studies measured social recovery outcomes (Table 2). One RCT (Fowler et al., 2018) captured time use, finding that SRT led to large and significant gains in time spent in structured activity at 9 months, with some evidence of gains too at 15 months once adjusted for missing data (Fowler et al., 2018). All other studies focused on more subjective self-rated social outcomes such as life satisfaction, with two studies reporting small to medium interventional effects (Gabrielsen et al., 2019; Hambridge, 2017). Qualitative studies provided some evidence for interventional benefits on social recovery (Gabrielsen et al., 2019; Gee et al., 2018; Walsh & Minor-Schork, 1997).

**Table 2:** Quantitative study results

First author (date)	Measure	Baseline to post-treatment						Post-treatment to final follow-up				
		Intervention M (SD), n (%)		Control M (SD), n (%)		Effect size (estimate), [95% CI]	Test, p value	Final follow-up duration	Intervention M(SD), n (%)	Control M(SD), n (%)	Effect size (estimate), [95% CI]	Test, p value
		Baseline	Post-intervention	Baseline	Post-intervention				Final follow-up	Final follow-up		
*Hope*												
Aubuchon-Endsley & Callahan, (2014)	SHS	-	N/A	N/A	N/A	N/A	N/A	N/A	N/A	N/A	N/A	N/A
Conklin (2009)	THS	43.8 (6.8), - (-)	-	43.9 (6.9), - (-)	-	N/A	N/A	N/A	N/A	N/A	N/A	N/A
	SHS	30.98 (6.76), 54 (100)	34.30 (5.40), 46 (85.1)	30.06 (7.03), 49 (100)	33.67 (6.77), 36 (73.5)	− 0.04 (d^a^, bg)	T = − 0.68, p = .50	N/A	N/A	N/A	N/A	N/A
Dowling & Rickwood, 2015	CHS-PTPB	2.39 (1.07), - (-)	2.56 (1.01), -(-)	N/A	N/A	-	-, ns	N/A	N/A	N/A	N/A	N/A
Fowler et al., (2018)	THS	34.6 (11.6), 66 (88.0)	36.5 (11.3), 54 (68.4)	35.5 (11.5), 67 (89.3)	36.13 (10.8), 46 (58.2)	2.21 (d, bg), [− 1.50, 5.93]	GMM, p = .24	15 months post-randomization	41.6 (10.4), 53 (70.7)	38.4 (9.9), 40 (50.6)	3.86 (d, bg), [− 0.27, 7.99]	GMM, p = .07
Gillig et al., 2019	SHS	5.80 (1.07), 56 (100)	6.23 (0.91), 56 (100)	N/A	N/A	0.54 (d^a^)	t = 3.87, p < .001	N/A	N/A	N/A	N/A	N/A
Green et al., (2007)	THS	43.86 (9.35, 25 (89.3)	49.63 (9.36), 25 (89.3)	43.96 (8.70), 24 (85.7)	39.74 (14.27), 24 (85.7)	1.08 (d^a^, bg)	F(1,35) = 6.65, p < .05	N/A	N/A	N/A	N/A	N/A
Isa et al. (2018)	CHS	17.44 (2.33), 18 (90.0)	29.78 (2.56), 18 (90.0)	N/A	N/A	1.99 (d^c^), [14.35, 23.09]^l^	T(17) = -15.74, p = .001	4 weeks post-intervention	30.72 (0.83), 18 (90.0)	N/A	0.39 (d^c,m^), [− 0.32, − 0.98]^l^	T(17) = 1.75, p = .10
Lin et al. (2013)	Hope scale(Al-Mabuk et al., 1995)	101.27 (15.17), 15 (78.9)	109.33 (16.28), 12 (70.6)	96.42 (13.88), 15 (78.9)	101.58 (20.28), 12 (70.6)	0.19 (d^a^, bg)	-, ns	8 weeks post-intervention	106.46 (18.15), 15 (78.9)	90.80 (21.54), 12 (70.6)	0.42 (d^a,m^, bg)	-, ns
Lin et al., (2014)	Hopefulness about the future scale	11.89 (2.99), 64 (100)	13.08 (1.88), 64 (100)	11.94 (3.01), 53 (88.3)	12.96 (2.31), 53 (88.3)	0.05 (d^c^, bg)	F = 0.05, ns (bg)	N/A	N/A	N/A	N/A	N/A
Ritschel et al., (2011)	CHS	16.80 (2.28), 6 (100)	57.67 (25.20), 6 (4.55)	N/A	N/A	2.6 (d^d^), [1.95, 6.36]	F(1,4) = 26.53, p < .001	N/A	N/A	N/A	N/A	N/A
Ritschel et al., (2016)	CHS	16.29 (4.98), 28 (100)	21^c^ (-), 21 (75.0)	N/A	N/A	0.98 (d)	t(20) = 4.02, p = .001	3- and 6-months post-baseline	25^e^ (-), 14 (50.0)	N/A	0.03 (d^f^), 0.66 (d^g^)	F(2,26) = 4.99, p < .05
Sælid & Nordahl, (2017)	HHI^h^	37.13 (3.26), 21 (100)	39.31 (4.23), 19 (90.5)	ATP = 38.02 (4.05), 21 (100) Inactive = - (-)	ATP = 40.11 (4.92), 17 (81.0), Inactive = - (-)	REBT = 0.58 (d, bg^i^), ATP = 0.12 (d,bg^j^)	F(3,102) = 5.99, p < .05	N/A	N/A	N/A	N/A	N/A
Smith et al., (2011)	HHS	68.79 (-), 34 (100)	73.26 (-), 33 (97.1)	Yoga = 68.07 (-), 15 (100), Inactive = 71.78 (-), 32 (100)	Yoga = 67.80 (-), 10 (66.7), Inactive = - (-), 0 (0)	1.79 (d^c^, bg)	F(4,134) = 3.81, p = .006	N/A	N/A	N/A	N/A	N/A
Teodorczuk et al., (2019)	CHS	25.86 (5.57), - (-)	22.50 (5.29), - (-)	21.87 (5.81), - (-)	24.07 (5.12), - (-)	-	-, p = 0.42	6 weeks post-intervention	24.14 (4.98), - (-)	22.40 (6.22), - (-)	-	-, p = 0.41
*Depression*												
Aubuchon-Endsley & Callahan (2014)	BDI-II	22.21 (11.54)	-	N/A	N/A	N/A	N/A	N/A	N/A	N/A	N/A	N/A
Conklin (2009)	BDI-II	16.50 (8.64), 54 (100)	9.83 (8.68), 47 (87.0)	18.77 (8.73), 49 (100)	12.64 (9.52), 36 (73.5)	− 0.06 (d^a^, bg)	T = − 1.25, p = .22	N/A	N/A	N/A	N/A	N/A
Fowler et al., (2018)	BDI-II	18.4 (11.6), 73 (97.3)	14.8 (12.8), 62 (78.5)	19.2 (12.2), 75 (100)	16.2 (11.3), 55 (69.6)	− 1.57 (d, bg), [− 4.84, 1.71]	GMM, p = .35	15 months post-randomization	14.0 (11.9), 55 (73.3)	12.2 (11.7), 465.3 (54.4)	0.75 (d, bg), − 3.26, 4.76]	GMM, p = .71
Gabrielsen et al., (2019)	HADS	8.5 (4.3), 32 (100)	9.0 (4.5), 31 (96.9)	N/A	N/A	0.12 (d^l^)	-, ns	12 months post-intervention	6.8 (4.4), 19 (59.4)	N/A	− 0.49 (d^m^)	-, p < .001
Gillig et al., 2019)	CES-D-4	2.70 (1.98), 56 (100)	1.79 (1.66), 56 (100)	N/A	N/A	− 0.52 (d^c^)	t = − 3.79, p < .01	N/A	N/A	N/A	N/A	N/A
Green et al., (2007)	DASS-21 depression	14.87 (11.33), 25 (89.3)	8.63 (11.86), 25 (89.3)	9.36 (6.80), 22 (78.6)	8.33 (7.77), 22 (78.6)	− 0.54 (d^a^, bg)	T = − 1.968, p < .05	N/A	N/A	N/A	N/A	N/A
Hambridge (2017)	DASS-21 depression	11.12 (9.76), 25 (100)	13.44 (11.56), 25 (100)	6.24 (8.6), 25 (100)	8.32 (11.44), 25 (100)	0.44 (d^c^, bg)	-, ns	N/A	N/A	N/A	N/A	N/A
Isa et al., (2018)	BDI	24.44 (11.18), 18 (90.0)	3.94 (2.10), 18 (90.0)	N/A	N/A	− 1.99 (d^c^), [− 4.82, − 2.65]^n^	t(17) = − 7.8, p = 0.001	4 weeks post-intervention	3.78 (1.31), 18 (90.0)	N/A	− 0.12 (d^c,m^), [− 0.71, − 0.59]^l^	t(17) = − 0.57, p = 0.001
	MFQ	13.33 (4.30), 18 (90.0)	2.83 (1.39), 18 (90.0)	N/A	N/A	− 3.30 (d^c^), [− 6.88, − 4.04]^n^	t(17) = − 8.69 p = 0.001	4 weeks post-intervention	2.22 (1.17), 18 (90.0)	N/A	− 0.36 (d^c,m^) [− 0.99, − 0.32]^l^	t(17) = − 0.72, p = 0.1
Leibovich et al., (2020)	BDI	20 (N/A), 1 (100)	2 (N/A), 1 (100)	N/A	N/A	-	N/A	N/A	-	N/A	-	N/A
	HDRS	14 (N/A), 1 (100)	6 (N/A), 1 (100)	N/A	N/A	-	N/A	N/A	-	N/A	-	N/A
Lin et al., (2013)	CES-D	23.00 (8.28), 15 (78.9)	16.80 (9.27), 12 (70.6)	25.25 (7.51), 15 (78.9)	18.83 (8.97), 12 (70.6)	− 0.02 (d^a^, bg)	-, ns	8 weeks post-intervention	17.08 (6.28), 15 (78.9)	19.70 (6.41), 12 (70.6)	− 0.11 (d^a,c^, bg)	-, ns
Lin et al., (2014)	CES-DC	40.96 (9.30), 64 (100)	38.89 (8.41), 64 (100)	36.82 (9.51), 53 (88.3)	35.53 (5.93), 53 (88.3)	− 0.40 (d^c^, bg)	F = 14.89, p < .001	N/A	N/A	N/A	N/A	N/A
Ritschel et al., (2011)	CDRS-R	57.67 (11.18), 6 (100)	27.67 (8.07), 6 (100)	N/A	N/A	− 0.39 (d^d^), [− 3.04, − 0.18]	F(1,5) = 19.94, p < .01	N/A	N/A	N/A	N/A	N/A
	BDI-II	28.00 (6.51), 6 (100)	6.00 (5.87), 6 (100)	N/A	N/A	− 7.27 (d^d^), [− 10.68, − 3.85]	F(1,5) = 330.00, p < .001	N/A	N/A	N/A	N/A	N/A
Ritschel et al., (2016)	CDRS-R	60.29 (10.24), 28 (100)	36^c^ (-), 21 (75.0)	N/A	N/A	− 0.63 (η^2^)	F(2,40) = 33.60, p < .001	6 months post-baseline	31^c^ (-), 14 (50.0)	N/A	− 0.06 (η^2m^)	-, p = .44
	BDI-II	27.84 (10.69), 28 (100)	14^c^ (-), 21 (75.0)	N/A	N/A	− 0.63 (η^2^)	F(2,40) = 34.14, p < .001	6 months post-baseline	11^c^ (-), 14 (50.0)	N/A	− 0.04 (η^2m^)	-, p = .59
Sælid & Nordahl, (2017)	HADS	12.47 (3.33), 21 (100)	7.21 (3.53), 19 (90.5)	ATP = 12.17 (3.43), 21 (100), Inactive = 11.70 (3.62), 20 (100)	ATP = 8.47 (4.00), 17 (81.0), Inactive = 10.60 (5.91), 20 (100)	REBT = − 1.53 (d), − 0.70 (d, bg^i^) ATP = − 0.99 (d), − 0.42 (d, bg^j^)	F(2,53) = 4.59, p < .05	N/A	N/A	N/A	N/A	N/A
Smith et al., (2011)	DASS-21 depression	12.79 (-), 34 (100)	7.94 (-), 33 (97.1)	Yoga = 10.71 (-), 15 (100), Inactive = 5.87 (-), 32 (100)	Yoga = 5.00 (-), 10 (66.7), Inactive = - (-), 26 (81.3)	− 1.86 (d^c^, bg^k^)	F(4,134 = 4.34, p = .002	N/A	N/A	N/A	N/A	N/A
Teodorczuk et al., (2019)	RCADS-SV	60.36 (12.45), - (-)	56.71 (13.36), - (-)	55.67 (9.06), - (-)	54.93 (10.74), - (-)	-	-, p = .70	6 weeks post-intervention	55.29 (11.53), - (-)	58.07 (18.15), - (-)	-	-, p = .63
*Social recovery*												
Dowling & Rickwood, 2015)	Life satisfaction (SWLS (Diener et al., 1985))	2.58 (0.78), - (-)	2.66 (0.85), - (-)	N/A	N/A	-	-, ns	N/A	N/A	N/A	N/A	N/A
Fowler et al., (2018)	Time spent in structured activity (TUS Hodgekins et al., 2015; Short, 2006))	11.0 (7.5), 75 (100)	26.6 (24.2), 73 (97.3)	12.0 (8.6), 79 (100)	18.0 (20), 70 (88.6)	8.08 (d, bg), [2.50, 13.66]	GMM, p = .005	15 months post-randomization	23.0 (19.0), 68 (90.6)	22.5 (23.3), 60 (75.9)	0.05 (d, bg) [− 5.15, 5.26]	GMM, p = .98
Gabrielsen et al., (2019)	Life satisfaction (SWLS (Diener et al., 1985))	16.4 (6.4), 32 (100)	15.2 (7.2), 31 (96.9)	N/A	N/A	-	-, ns	12 months post-intervention	18.4 (8.5), 19 (59.4)	N/A	0.41 (d^m^)	-, p < .05
	Life effectiveness (LEQ-H (Neill et al., 2003))	4.2 (1.5), 32 (100)	4.4 (1.4), 31 (96.9)	N/A	N/A	0.14 (d^l^)	-, ns	12 months post-intervention	5.0 (1.0), 19 (59.4)	N/A	0.49 (d^m^)	-, p < .001
Hambridge (2017)	Life satisfaction (BMSLSS (Bickman et al., 2010))	2.36 (0.81), 25 (100)	3.56 (0.79), 25 (100)	4.02 (0.77), 25 (100)	3.85 (0.90), 25 (100)	0.34 (d^c^, bg)	-, ns	N/A	N/A	N/A	N/A	N/A
Leibovich et al., (2020)	Quality of life (Q-LES-Q (Endicott et al., 1993))	3.13 (N/A), 1 (100)	3.44 (N/A), 1 (100)	-	N/A	-	N/A	N/A	-	N/A	-	N/A

*SHS* State Hope Scale (Snyder et al., 1996), *THS* Trait Hope Scale (Snyder et al., 1991), *CHS-PTPB* Children’s Hope Scale-Peabody Treatment Progress Battery (Dew-Reeves et al., 2012), *CHS* Children’s Hope Scale(Snyder et al., 1997), *HHI* Herth Hope Index (Herth, 1992), *HHS* Herth Hope Scale (Herth, 1991), *BDI-II* Beck Depression Inventory II (Beck et al., 1996), *HADS* Hospital Anxiety and Depression Scale (Zigmond & Snaith, 1983), *CES-D-4* 4-item CES-D (Melchior et al., 1993), *DASS-21 depression* Depression, Anxiety and Stress Scales (depression subscale) (Lovibond & Lovibond, 1995), *BDI* Beck Depression Inventory (Beck et al., 1961), *MFQ* Mood and Feelings Questionnaire (Angold et al., 1995), *HDRS* Hamilton Depression Rating Scale (Hamilton, 1967), *CES-D* Center for Epidemiologic Studies Depression Scale (Radloff, 1977), *CES-DC* Center for Epidemiologic Studies Depression Scale for Children (Fendrich et al., 1990), *CDRS-R* Children's Depression Rating Scale-Revised (Poznanski et al., 1984), *RCADS-SV* Revised Child Anxiety and Depression Scale-Short Version (Ebesutani et al., 2012). Effect sizes are within groups unless otherwise labelled as bg = between-groups. d = Cohen’s d. ns = not significant. η^2^ = partial eta squared. GMM = Generalized Mixed Models. N/A = Not applicable. - = not reported and could not be otherwise calculated

^a^Calculated using formulae (Morris, 2008)

^b^Calculated using formula from (Morris & DeShon, 2002) using an estimated pre-post correlation of 0.525 calculated from provided pre-follow-up and post-follow-up effect sizes, M and SD (Gabrielsen et al., 2019)

^c^Not supplied by authors, calculated using formula(Field, 2013) with published M, SD and r)

^d^Calculated using formula (Morris & DeShon, 2002) using reported M and SD, and r calculated from raw data (Ritschel et al., 2011)

^e^Not supplied by authors, estimated from presented graphs

^f^Post-treatment to 3-month follow-up (first follow-up)

^g^3-month follow-up to 6-month follow-up

^h^Measured first session onward

^i^Rational emotive behavior therapy (REBT) versus control only

^j^Attentional placebo control (ATP) versus control only

^k^Yoga and meditation versus yoga only

^l^Calculated using formula (Morris & DeShon, 2002) with an estimated pre-post correlation of 0.485 calculated using provided pre-follow-up and post-follow-up effect sizes, M and SD (Gabrielsen et al., 2019)

^m^Post-treatment to final follow-up

^n^Calculated based on formula (Lenhard & Lenhard, 2016) using reported M and SD

##### Standard Mental Health Care

Eight studies presented qualitative (see Online Appendix F) experiences of standard mental health care in the UK (Bury et al., 2007; Gee et al., 2018), Canada (Davidson, 2008; Leavey, 2005), Australia (Rayner et al., 2018), USA (Davidson, 2008), Norway (Binder et al., 2013) and Germany (Weitkamp et al., 2017). Four studies focused on a single intervention or psychotherapy (Binder et al., 2013; Bury et al., 2007; Davidson, 2008; Weitkamp et al., 2017) and three on any previous service experiences (Gee et al., 2018; Leavey, 2005; Rayner et al., 2018). Positive therapeutic relationships, described by one participant as a “bond of hope”, were described as providing motivation, inspiration and as catalyzing positive change (Davidson, 2008). It was important that the professional was hopeful, both wanting positive outcomes for the young person and believing in their likely occurrence (Davidson, 2008). Experience of “venting” in early therapy mobilized hopefulness and positive expectancies of functional improvement (Davidson, 2008). Therapist assessment, when performed competently and with relational authenticity, enhanced hopefulness through connection with the young person’s uniqueness and strengths, whilst also bringing order to the felt sense of chaos and hopelessness (Binder et al., 2013). Using standardized assessment tools was validating and normalizing; providing hopefulness that the existence of standardized frameworks to capture problem experiences might be indicative of the existence of solutions (Binder et al., 2013). Conversely, participants in one study experienced UK youth mental health service provision as too limited, but some suggested this encouraged them to exert self-agency in their recovery (Gee et al., 2018).

One study quantitatively assessed experiences of an online counselling service (Dowling & Rickwood, 2015) (Table 2). There was no significant effect of one or two sessions on hopefulness, irrespective of observed session progress or depth, although there was a significant reduction in psychological distress (Dowling & Rickwood, 2015). Similarly, in the trial of Social Recovery Therapy (Table 2), standard Early Intervention in Psychosis services led to reductions in depression, but no significant gains in hopefulness (Fowler et al., 2018). The lived experience panel emphasized that mental health services can enhance hopefulness. However, the panel described services as focusing too little on hopefulness and that it should be considered a “*first resort*”.

#### In What Settings and Contexts, and For Whom, Does Hopefulness Appear Most Important and Effective (Research Question 2)?

There was no clear evidence of hopefulness being differentially important or beneficial in different contexts. Effect sizes did not seem to observably differ consistently according to study sample (e.g. size, age, gender, population, baseline hopefulness or depression severity) or intervention characteristics (e.g. type, duration, session number, or mode). Qualitative studies positioned hopefulness as generally important to recovery in depression, saying that hopefulness is important to hopeless people (Walsh & Minor-Schork, 1997). For adolescents aged around 18 years, impending adulthood appeared to provide motivation to engage in psychotherapy (Gee et al., 2018); yet studies did not indicate lesser importance or effects related to hopefulness at other ages. One study suggested that imagining the best possible future self can be challenging or perceived negatively (Teodorczuk et al., 2019). Another study found a significantly greater degree of self-reported goal completion and significantly smaller reduction in depression for people who had greater baseline trait hopefulness; but only in the active goal-skills intervention and not the goal-visualization control (Conklin, 2009). Therefore, goal-skills interventions may better enhance hopefulness for hopeful people but have less impact on depression.

The lived experience panel agreed that whilst hopefulness is important to all youth with depression, it is not necessarily unilaterally beneficial. In general, identified challenges to hopefulness included difficulties in feeling agentic and the sense that repeated failures to achieve goals may erode hopefulness over time. Some youth spoke of hopefulness becoming salient during adversity, rather than being an underlying or continuous presence. With respect to specific barriers, it was suggested that locating and enacting hopefulness is arguably more challenging for people with severe or long-lasting depression and in the context of additional intersecting challenges (Online Appendix G); “*people who need hopefulness tend to be faced with actions that bar them from hope*”.

#### What are the Putative Processes and Mechanisms by Which Hopefulness Impacts on Outcomes (Research Question 3)?

##### Where Hopefulness Comes from and What Youth with Depression Hope For

Five qualitative (Anttila et al., 2015; Bury et al., 2007; Midgley et al., 2016; Watsford et al., 2013; Weitkamp et al., 2017) and one quantitative (Aubuchon-Endsley & Callahan, 2014) study focused on hopes or expectancies for intervention or outcome. Two studies related to a trialed novel intervention (Anttila et al., 2015; Midgley et al., 2016) and four to standard psychotherapy (Aubuchon-Endsley & Callahan, 2014; Bury et al., 2007; Weitkamp et al., 2017) or mental health care more generally (Watsford et al., 2013). Three studies captured hopes at intervention outset (Anttila et al., 2015; Midgley et al., 2016; Watsford et al., 2013) and three involved a more current or retrospective focus (Aubuchon-Endsley & Callahan, 2014; Bury et al., 2007; Weitkamp et al., 2017). Hoped-for changes appeared typical and normative, including increased self-understanding, independence, better coping, greater interpersonal relationship quality and quantity, positive engagement and performance in meaningful occupational and vocational activities (Anttila et al., 2015; Midgley et al., 2016; Weitkamp et al., 2017). Psychological hopes, e.g. greater self-understanding, were positioned as the foundation for and route to achieving positive social recovery outcomes such as better school performance (Midgley et al., 2016; Rayner et al., 2018). Youth’s hopes for their therapist echoed the qualities experienced as hope-enhancing in other studies; competent, experienced, professional, understanding, caring and nice (Midgley et al., 2016; Weitkamp et al., 2017).

Studies suggested that hopefulness was self-reinforcing in that the initial development of hopefulness within an intervention appeared to act as a primer for a chain of events in which youth became aware that the ongoing effortful pursuit of goals (Gabrielsen et al., 2019; Gee et al., 2018; Hambridge, 2017; Metsäranta et al., 2019). The associated observable small gains made further increased their hopefulness and provided motivation to pursue more ambitious goals (Gabrielsen et al., 2019; Gee et al., 2018; Hambridge, 2017; Metsäranta et al., 2019). A few studies considered how interventional components influenced hopefulness. Qualitative studies suggested that engaging in help-seeking itself built self-efficacy and resilience (Rayner et al., 2018), having a computer character personifying hope generated hopefulness (Shepherd et al., 2018), visually depicting one’s future self-image provided a “springboard” into actively expecting and planning for a positive future (Walsh & Minor-Schork, 1997), and that CBT techniques formed “building blocks” from which youth could use their increased self-agency to pursue meaningful goals (Gee et al., 2018). One quantitative study found that greater self-reported goal progress during the intervention predicted greater increase in hopefulness from post-intervention to one-week follow-up (Conklin, 2009).

Other studies focused on the relational enhancement of hopefulness. One study predicted that a LGBTQ camping intervention increased hopefulness through campers’ aspirational identification with camp counsellors and other campers, and associated positive identity formation and empowerment (Gillig et al., 2019); however this model was not empirically supported. However, individual hopefulness-enhancing interventions appeared predicated on a positive therapeutic relationship. Such relationships needed to be with professionals who held hopefulness for and cultivated it in youth (Davidson, 2008; Gee et al., 2018; Hambridge, 2017), through providing support (Rayner et al., 2018) and unconditional positive regard (Davidson, 2008; Hambridge, 2017), offering interpretations suggestive of potential for change (Leibovich et al., 2020), focusing on collaboratively identified meaningful goals (Gee et al., 2018), modelling and supporting the process of breaking down goals into small steps (Hambridge, 2017), finding solutions to barriers (Hambridge, 2017), helping youth achieve specific goals (Hambridge, 2017; Rayner et al., 2018), and continuing to embody hopefulness despite any setbacks (Davidson, 2008). One study participant emphasized that hopefulness arises from the therapist seeming strong and stable, for this communicates that problems are bearable and can be overcome (Weitkamp et al., 2017). Studies of specific psychological therapies and standard mental health care both suggested a benefit to ‘groupiness’ (Leavey, 2005; Pingitore & Ferszt, 2017; Walsh & Minor-Schork, 1997), with a psychotherapy group being termed a “gathering of hope” (Pingitore & Ferszt, 2017). Group benefits appear to hinge on common experience, collective agency and a shared goal of recovery (Leavey, 2005; Pingitore & Ferszt, 2017), discussing the future with other youth (Walsh & Minor-Schork, 1997), and enacting hopefulness through helping and supporting other group members (Pingitore & Ferszt, 2017).

Finally, whilst some youth described hopeful thinking as an intrinsic or adopted attitude (Gee et al., 2018; Leavey, 2005), other studies suggested its development is gradual. Two novel intervention trials found no post-intervention increase in hopefulness, but observed significant gains at 12 (Gabrielsen et al., 2019) and 15-month follow-up (Fowler et al., 2018). Another study found significant gains in hopefulness from 3 to 6-month follow-up (Ritschel et al., 2016). This pattern was not evident for depression, as follow-up effect sizes were observably smaller than at post-intervention (Table 2). Qualitative studies echoed a pattern of delayed increase in hopefulness. Hopefulness could be completely absent for youth at intervention outset (Hambridge, 2017), in the context of depression involving diminished ambition and interest in life (Anttila et al., 2015; Metsäranta et al., 2019; Walsh & Minor-Schork, 1997) and compounded by others’ low expectations for the young person’s future (Hambridge, 2017). Youth stated that interventions need to be long-term (Gabrielsen et al., 2019; Gee et al., 2018), for meaningful changes would take months or potentially years to be noticeable (Gabrielsen et al., 2019). Thus, hopefulness may need to be gradually built through an evolving sense of therapeutic gains (Hambridge, 2017; Metsäranta et al., 2019) within a supportive and encouraging interpersonal environment (Hambridge, 2017). Hopefulness gained during intervention can then function as a primer and motivator for ongoing and increasingly effortful goal pursuit post-intervention (Gabrielsen et al., 2019; Gee et al., 2018; Hambridge, 2017).

The lived experience panel described hopefulness as unique and individual with respect to its sources, nature, and effects, stating it “*will never be one size fits all”*. The panel agreed that positive therapeutic relationships between professionals and youth are necessary for scaffolding hopefulness. The group emphasized that mental health professionals should not be too “*explicit*” or directive in discussing or encouraging hopefulness; “[t]*herapists should work to find what uniquely brings their patient hope, rather than trying to be prescriptive about it*”. The panel advised professionals to try and implicitly “*trigger*” hopeful thinking through providing validation, empathy and authenticity, forming meaningful connection with youth’s unique hopefulness, using sensitivity and gentleness, providing support to identify meaningful current and short-term goals, and helping to break down goals into smaller parts (see Table 3).

**Table 3:** Youth lived experience panel quotes about how mental health professionals can enhance hopefulness

Component	Illustrative quotation
Validation, empathy and authenticity	*“I’ve had experiences where therapists and people in mental health mentoring roles have encouraged me to try and feel hope about situations that are genuinely extremely negative. It can make me feel like I haven’t been listened to, like they haven’t understood the extent of the situation, or like they’re trying to put a plaster over some glaring societal issues which are linked to probably the majority of mental health issues in the population.*”
Connect with unique hopefulness	*“Sitting down with young people getting to know them and supporting them in their own unique ways of finding hope.”*
Sensitive and gentle triggering of hopeful thinking processes	*“…mental health professionals* [should] *not be too explicit during therapy *etc. *about hopefulness as I think this could potentially make people feel worse if they can’t think of anything they feel hopeful about, but instead if the professionals address it in a non-direct* [way]*, they are more likely to get some answers out of people as to what they feel hopeful about without the person even realizing it, then they can build on helping the person to recognize that this is there thing(s) to be hopeful about.”* *“…being told you need to have hope doesn't help. If you’re feeling depressed maybe you don’t feel like you have many good things in life. But triggering the thought process about what you can have hope for.”*
Support to identify meaningful current and short-term goals	*“I agree that if a* [professional] *simply identifies things/goals to be hopeful for, this isn’t enough for the* [young person]*, they need to believe in the goals and need support to achieve them. For a* [young person] *experiencing low mood/depression, they may need goals to focus on in the next days/weeks as longer-term goals may be overwhelming. For example, if a* [professional] *suggests being hopeful about the possibility of future careers and relationships, this could increase anxiety for some people and could be counterproductive. I think that it would be more useful to focus on what’s important for that individual in that point in time.”*
Helping to break down goals into smaller parts	*“I think it helps to emphasize the power of small actions. This allows the young person to trust in the process because often goals can take time to achieve and they are built on repeating small actions over time. A small action is often more accessible so the young person can still feel in control.”*

##### Putative Mechanisms of the Impact of Hopefulness

**Fig. 3:**
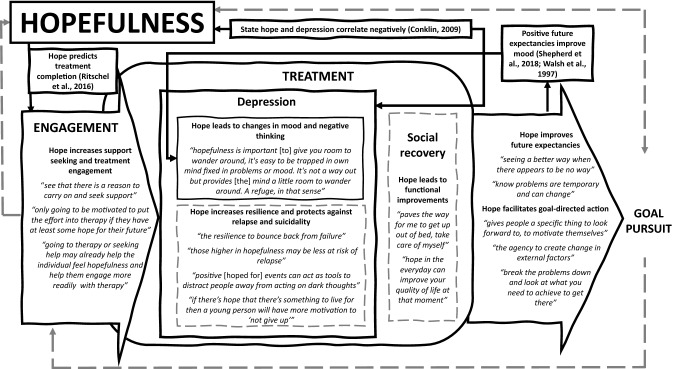
Preliminary process model of the mechanistic impact of hopefulness on clinical and social recovery outcomes for youth with depression. *Notes*: Grey dashed lines indicate putative mechanisms identified by lived experience experts (verbatim quotes in italics). Black solid lines indicate putative mechanisms identified in reviewed scientific evidence

Evidence regarding mechanisms of hopefulness was very limited. Two quantitative studies reported associations between pre-treatment hopefulness or expectancies and engagement (Aubuchon-Endsley & Callahan, 2014; Ritschel et al., 2016); with greater hopefulness predicting the likelihood of completing behavioral activation (Ritschel et al., 2016), but more positive treatment expectancies predicting reduced university counselling attendance (Aubuchon-Endsley & Callahan, 2014). The latter study found no correlation between hopefulness and treatment expectancies (Aubuchon-Endsley & Callahan, 2014), however, suggesting these may reflect different phenomena. Two quantitative studies considered mechanisms the impact of hopefulness. One found a concurrent association between increased state hopefulness and reduced depression (Conklin, 2009) and the other hypothesized, but did not find, that the baseline level and gains in hopefulness would moderate the reduction in depression (Gillig et al., 2019). Multiple putative candidates, however, for how hopefulness impacts further outcomes were identified by lived experience experts. Their reported observations were that hopefulness facilitates support-seeking, improves mood and negative thinking, protects against relapse and suicidality, and motivates goal-directed action. Research evidence and lived experience identified potential mechanisms were combined into a preliminary hopefulness process model (Fig. 3), which suggests an ultimately self-reinforcing impact of hopefulness through increasing treatment engagement, clinical and functional improvements, and ongoing goal pursuit.

#### Risk of Bias Across Studies

With respect to the risk of bias at the outcome level, there is moderate certainty in the review conclusions according to GRADE (Oxman, 2004) domains. Publication bias was not estimated. There is a high risk of bias with respect to the variable quality of individual studies. However, there appears to be low inconsistency and indirectness, for almost all interventions appeared to improve hopefulness and reduce depression. All were evaluated within the population of interest whilst simultaneously reflecting diversity in age, gender, ethnicity, geography and setting. Most studies, irrespective of quality, reported moderate to large effect sizes on hopefulness and depression, however, whilst confidence intervals were infrequently reported or calculable, those present suggest low precision. Nonetheless, there is a high level of coherence across quantitative, qualitative, and mixed methods evidence and with the perspectives of young lived experience experts consulted.

## Discussion

Hopefulness is arguably of central importance to the recovery of youth with major or complex youth depression, yet understandings are limited as to how hopefulness can best be enhanced in treatment and how it impacts on symptom and social recovery outcomes. Existing reviews have concluded that hopefulness predicts mental health and positive functioning generally for students (Griggs, 2017) and adolescents (Esteves et al., 2013), yet have not specifically considered evidence for the role and impact of hopefulness for youth with mental health problems. However, hopefulness is especially relevant to depression, which is characterized by negative thoughts and expectations for the future (Bjärehed et al., 2010). Arguably hopefulness may underlie all positive psychotherapeutic change (Taylor, 2000), whether explicitly a focus of therapy or not. Hopefulness is additionally important more broadly in standard mental health treatment. Yet limited research has focused on evidence for benefits of hopefulness in these different treatment settings, or considerations of how to best enhance hope and how hope in turn impacts on symptomatic and social recovery. This review synthesized evidence of the development and impact of hopefulness within specific psychological interventions and standard mental health care in youth depression.

The conclusion of this review is that hopefulness is a key active ingredient for youth with major or complex depression. The evidence review suggests that standard mental health care and varied novel CBT-based and alternative talking and activity-based interventions appear able to effectively engender hopefulness and reduce depression. Social Recovery Therapy (Fowler et al., 2018; Gee et al., 2018) and behavioral activation (Ritschel et al., 2011, 2016) reflected higher quality and multiple study evidence and thus maybe be of more reliable benefit. Camping, integrated yoga and meditation, life-coaching and a brief goal-skills intervention additionally appeared effective in enhancing hope and reducing depression (Conklin, 2009; Gabrielsen et al., 2019; Gillig et al., 2019; Green et al., 2007; Smith et al., 2011). Therefore, the most promising specific interventions are those characterized by a clear focus on goals and which use a behavioral therapy or activity-based approach.

In all but one case in which specific psychological therapies failed to significantly increase hopefulness relative to their controls, this was due to increased hopefulness in the control conditions. Therefore, it seems that the simple experience of being heard, for example in an assessment with a competent, warm, hopeful and authentic assessor (Anttila et al., 2015; Binder et al., 2013; Davidson, 2008; Midgley et al., 2016), can be sufficient to start engendering hopefulness in youth depression. Whilst the majority of interventions, including an active listening control (Sælid & Nordahl, 2017) appeared hope-enhancing, hopefulness did not increase following yoga-as-exercise (Smith et al., 2011) or two online counselling sessions (Dowling & Rickwood, 2015). Thus, effective hopefulness interventions may need to be imbued with relational intimacy and personal meaning. Similarly in standard mental health care, relational qualities were central to enhancing hopefulness, either in engagement with a competent, caring, relationally authentic professional, or within a group of youth with shared experiences (Binder et al., 2013; Bury et al., 2007; Davidson, 2008).

Notably a brief positive psychology intervention (Teodorczuk et al., 2019) was not associated with improvement in hopefulness or depression. This intervention used a large number of foci and tasks, with two activities reported as unhelpful or even harmful; practicing acts of kindness, which appeared to result in the participant being mocked by others and imagining the positive “future self” which was challenging for a participant. The challenge of imagining the “future self” appeared to be related to defensive pessimism, i.e. the self-protective avoidance of positive expectancies; a strategy that similarly appeared evidently in use among youth in two of the reviewed qualitative studies (Hambridge, 2017; Weitkamp et al., 2017). Interventionists must therefore be sensitive to the system around the young person, for it may be hostile and characterized by low support and pessimistic future expectations (Gee et al., 2018; Hambridge, 2017; Teodorczuk et al., 2019).

## Limitations

This review is limited by the fact that study evidence was of variable and often poor quality. As the lower quality studies appeared to report greater effect sizes for reduction in depression, these effects must be taken cautiously. However, study quality has limited bearing on the conclusions about hopefulness. There was no clear relation between quality and effect sizes and lower quality qualitative studies contributed more peripherally; mainly referring to hopes for psychotherapy. The lack of process evaluation, experimental and dismantling approaches in the reviewed research evidence limits somewhat conclusions regarding the best interventions, with what elements, for enhancing hopefulness and how, or what constitutes an adequate treatment “dose”. Moreover, this review used a narrative synthesis approach. The limitations of this approach are that effect sizes were compared descriptively, not statistically synthesized. The lack of statistical synthesis, and the inclusion of grey literature, which by its nature involves a more complex, less systematic searching process, may undermine the reproducibility of the current study relative to more traditional systematic reviews (Mahood et al., 2014) and meta-analyses (Campbell et al., 2020).

## Strengths

The current study provides a rich understanding of the enhancement and impact of hopefulness in youth depression. The incorporation of lived experience insights in this review is a key strength of the work. The lived experience panel both contributed to the research team’s interpretation of the systematic review and narrative synthesis findings, and generated their own responses to the research questions. The latter in particular contributed unique and valuable insights, especially regarding the processes by which hopefulness can be enhanced, which were not apparent from the published or grey literature as it stands. Such benefits are very much in keeping with those observed in previous participatory style reviews (Harris et al., 2016). Moreover, the inclusivity of the current approach, with respect to synthesizing both scientific and grey literature, and quantitative, qualitative and mixed methods research, broadens the scope of the evidence reviewed (Mahood et al., 2014) and can be considered a strength of this work. The current review did not focus on COVID-19 or its sequelae, and synthesized research was conducted before the global pandemic, yet hopefulness has particular relevance for young people post-pandemic (YoungMinds, 2020); protecting against the mental health impacts of the pandemic (Gallagher et al., 2021) and long periods of social restrictions (Hu et al., 2021). Current findings will have resonance for mental health professionals in effectively supporting young people with their mental health recovery in the post-pandemic context.

## Clinical and Research Implications

The key clinical implications of this review are that hopefulness appears to be an important target for intervention for youth with major or complex depression. Hopefulness can be enhanced within standard mental health care and in the provision of specific psychotherapeutic interventions. Interventions need to offer a positive relational environment through individual mentor and/or therapeutic relationships and access to groups of youth with similar experiences (Pingitore & Ferszt, 2017; Walsh & Minor-Schork, 1997). Professionals encountered should be competent, authentic and communicate hopefulness and unconditional positive regard (Binder et al., 2013; Davidson, 2008; Hambridge, 2017; Midgley et al., 2016). The core interventional tasks appear to be the collaborative setting of personally-relevant goals, engagement in meaningful activity, and scaffolding hopefulness and positive expectancies for goal attainment. The processes of supporting goal identification and progress can be enhanced by drawing on hope theory (Snyder & Taylor, 2000) and the research evidence presented here; especially with regard to the lived experience mandate to not be directive or prescriptive in attempts to enhance hopefulness. Longer-term and systemic intervention may be needed, especially for people with complex difficulties (Fowler et al., 2019), to gradually build and sustain hopefulness both for the young person and in the wider system. Moreover, whilst youth did hope for psychological change, many hopes and goals related to social recovery. Therefore, professionals should be poised to support youth to enhance their self-agency and pathways thinking across social and occupational life domains, and to use activity and behavioral therapy approaches in their work.

The current study supports a developmental science perspective on hopefulness as an individual level variable that influences and is influenced by the context or ecology around the adolescent (Schmid & Lopez, 2011). In addition, the study adds two important further considerations. First, hopefulness appears to be reduced in the context of complexity, i.e., comorbid mental health problems, social identities and access to support and resources. Secondly, hopefulness influences the degree to which youth engage in relationships and activities which could become hopefulness-inducing, i.e., hope influences mental health help-seeking and treatment engagement. These are important considerations as the developmental science of hopefulness is under-studied especially with reference to diverse and vulnerable youth (Schmid & Lopez, 2011). Current findings suggest that whilst the “objects” of hopefulness, i.e., the desired future goals, for youth with depression are normative and comparable to adolescents in general (Nurmi, 1991), the process of engendering hopefulness may be more challenging, especially in the context of complexity. Current findings which suggest hope can be increased outside of clinical treatment settings are especially helpful in this regard, as educators and others in the wider surrounding system can offer a hopeful environment which may be missing from the family context. Educators and others can use insights generated in this review, as to the need to gently encourage hopeful thinking in the context of meaningful engagement, ideally involving peers, and with focus on personally-relevant goals. The provision of this hopeful environment should begin the hope-engendering process, which itself will facilitate help-seeking and treatment engagement for youth who need more specialist mental health support. Moreover, the current study furthers understandings of how hopefulness helps adolescents to construct positive ideas and expectations of their future (Schmid et al., 2011) in the context of depression. One lived experience panel member’s experience of hopefulness as providing the “mind a little room to wander around” (see Fig. 3) may be a phenomenological manifestation of hopefulness compensating (Sears, 2007) for executive functioning problems observed in adolescence and depression (Luciana, 2016). It must be acknowledged nonetheless, that the current study synthesized findings from studies spanning a large age period (14–25 years) and further research is still needed to consider the impact and enhancement of hopefulness specifically within more narrowly-defined developmental stages (Griggs & Walker, 2016).

A key implication for policy-makers and commissioners is to consider how best to structure and fund treatment services in supporting professionals’ own hopefulness and positive outcome expectancies. Professional hopefulness and expectancies facilitate patient hopefulness (Coppock et al., 2010) and positive clinical and social recovery outcomes (Berry & Greenwood, 2015; O’Connell & Stein, 2011), perhaps irrespective of interventions provided (Young et al., 1998). Hopefulness appears to have broad transdiagnostic relevance to treatment engagement and recovery (Schrank et al., 2012), including in anxiety (Gallagher et al., 2020) and psychosis (Berry & Greenwood, 2015), and in physical illness (Griggs & Walker, 2016). Therefore, enhancing hopefulness in health service systems clearly aligns with the clinical and diagnostic complexity typically seen in youth (Hickie et al., 2013). Evidence suggests professional hopefulness can be increased through services being recovery-oriented (Niebieszczanski et al., 2016), providing support for coping and managing stress (Larsen et al., 2013), hopeful supervision (Collins, 2015), and regular reflections on beliefs about the therapeutic use of hopefulness (Larsen et al., 2013).

The lived experience panel generated and ranked their top ten future research priorities (Table 4). Important additional priorities include conducting high-quality trials, with long follow-ups and in-built process evaluations to better understand how hopefulness grows and influences clinical and social recovery. Observational and experimental dismantling studies could clarify the necessary and sufficient intervention conditions to improve hopefulness and depression. Such studies could additionally be used to identify the best implicit and explicit relational practices professionals can use to scaffold hopefulness in youth mental health settings. The widespread inclusion of a hopefulness measure in interventional studies in youth depression would facilitate increased intra- and cross-study understandings of its psychotherapeutic qualities. The Trait Hope Scale (Snyder et al., 1991) is recommended as a brief measure which captures dispositional hopefulness; that which underlies more momentary or specific hopes but remains amenable to intervention.

**Table 4:** Young lived experience expert generated top ten priorities for future research investigating hopefulness as a key active ingredient for youth with major or complex depression

Priority ranking	Research priority
1	*How do specific marginalizations, for example socioeconomic status, race, sexuality, and gender identity, interact with how youth with depression experience hopefulness?*
2	*How does having hopefulness benefit a young person’s ability to cope with depression compared to people who do not have hopefulness or do not understand hopefulness as motivational or goal-directed?*
3	*How can professionals better help in triggering more hopefulness for youth who have long-term depression (including with complex difficulties)?*
4	*How can hopefulness be promoted in the transition from child/youth to adult mental health services?*
5	*How can people surrounding a young person with depression help to encourage growth in hopefulness?*
6	*How can the importance of hopefulness and goals be made clear to youth with depression?*
7	*How can teaching self-advocacy affect hopefulness in children or youth with depression?*
8	*What types of group therapy or support groups are best for promoting hopefulness in recovery for youth with depression?*
9	*Are there differences in the benefits of hopefulness for youth with mixed anxiety and depression compared to solely depression?*
10	*How can encouraging community and/or political involvement increase hopefulness for children and youth with depression?*

## Conclusion

Hopefulness is important to positive functioning in adolescence, and especially in the recovery of youth with major or complex depression. The current study builds the limited existing understandings of how hopefulness is best enhanced in different treatment settings and how it impacts on important treatment outcomes. The findings of this systematic review and lived experience panel evidence synthesis show that hopefulness can be enhanced in individual and group interventions, brief and longer-term, across health, community and educational settings. Hopefulness facilitates treatment-seeking and engagement and appears self-reinforcing. Specialist intervention is not always needed; hopefulness can be first scaffolded in non-clinical settings. More work is needed to further clarify what works best for whom and when, but current findings emphasize the importance of positive relationship with a professional and/or other youth, the gentle triggering of hopeful thinking, and the identification and pursuit of personally meaningful goals.

## Supplementary Information

Below is the link to the electronic supplementary material.

Supplementary file1 (DOCX 86 kb)

## Data Availability

There are no new data associated with this manuscript.

## Declarations

### Preregistration

All research questions, methods and planned analyses used in this review were pre-registered in the review protocol 14/07/2020 (PROSPERO CRD42020192701).
