# Supplementary material for: A Systematic Review and Lived-Experience Panel Analysis of Hopefulness in Youth Depression Treatment
Source: Adolesc Res Rev. 2021 Jul 6;7(2):235–66. doi: 10.1007/s40894-021-00167-0 (PMC8260023; doi:10.1007/s40894-021-00167-0)
Supplement: Supplementary file 1 — Supplementary file1 (DOCX 86 kb) [file 40894_2021_167_MOESM1_ESM.docx]

**Appendices**

**Appendix A: Database search terms**

| Element | Search terms and Boolean operators |
| --- | --- |
| Sample age | (youth OR young OR adolescen* OR Child* OR student OR undergraduate OR pupil) AND |
| Phenomenon of interest | hope* AND |
| Sample condition | depress* AND |
| Research setting | (therap* OR psychotherap* OR intervention OR treatment OR "mental health service" OR "youth service" OR "psychology service" OR practice OR program* OR "positive youth development" OR strateg* OR coaching OR "residential care" OR "social care" OR "social work") AND |
| Research design | (trial OR pilot OR feasibility OR "pre-post" OR qualitative OR observational OR interview OR "focus group" OR survey OR experimental OR outcome OR follow-up) |

*Notes:* Where possible, results were restricted to English language (ASSIA, PsychArticles, PsychInfo, Web of Science), peer-reviewed publications (ASSIA, PsychoArticles, PsychInfo), and adult or young adult and adolescent samples (PubMed/Medline, PsychArticles). References of existing reviews of hopefulness for young people and/or clinical populations were screened (Bolier et al., 2013; Cutcliffe & Koehn, 2007; Esteves et al., 2013; Griggs, 2017; Griggs & Walker, 2016; Koehn & Cutcliffe, 2007; Loveday et al., 2018; Marques & Lopez, 2014; Sælid & Nordahl, 2017; Schrank et al., 2012, 2008b; Shek et al., 2017; Sin & Lyubomirsky, 2009; Venning et al., 2009; Weis & Speridakos, 2011). References of all included articles were additionally screened.

**Appendix B: Study inclusion criteria informed by SPIDER (Cooke et al., 2012)**

| Element | Inclusion criteria |
| --- | --- |
| Sample | 1. At least 50% of the sample were aged 14 to 25 years or in which the mean, median, or modal sample age was within this range 2. At least 50% of participants met depression caseness, operationalised as one or more of 2.i to 2.iii   2.i. Confirmation that at least 50% of participants had been given a diagnosis of depression using a diagnostic instrument  2.ii. Reported depression measure mean or median scores which exceeded published clinically relevant thresholds or in which at least 50% of participants scored in excess of these thresholds  2.iii. In recognition that many qualitative studies do not present diagnoses or explicit frequencies thereof, qualitative studies which reported a clinical sample with diagnoses explicitly described as including depression were included   1. As we were interested in major and complex depression (i.e. comorbid or co-occurring depression in the context of other mental health diagnoses), studies focused on participants with diagnoses additional to depression were included |
| Phenomenon of Interest | 1. Quantitative studies with a focus on a specific psychological intervention or standard mental health care that used a measure of hopefulness 2. Qualitative studies with a focus on a specific psychological intervention or standard mental health care that collected data or presented themes or subthemes which pertained to hopefulness 3. Mixed methods studies needed to satisfy criteria 4 and/or 5 |
| Design | 1. Randomised Controlled Trial, uncontrolled pre-post interventional, or observational (including qualitative) designs 2. Any health, educational or community setting and within any geographic locality |
| Evaluation | 1. Quantitative studies needed to use a measure of depression or a social recovery outcome 2. Qualitative studies needed to collect data or present themes or subthemes on hopefulness as a component or outcome of specific psychological intervention or standard mental health care 3. Mixed methods studies needed to satisfy criterion 9 and/or 10 |
| Research | 1. Quantitative, qualitative and mixed methods studies, including case studies |

**Appendix C: Measurement of hopefulness**

| Measure | Type of hopefulness | Conceptual model | Number of items | Example items | Use in studies reviewed |
| --- | --- | --- | --- | --- | --- |
| Children’s hope Scale (CHS)(CR Snyder, 1997) | Trait or dispositional | Snyderian cognitive | 6 | - *I am doing just as well as other kids my age* (self-agency subscale) - *I can think of many ways to get the things in life that are most important to me* (pathways subscale) | 4 |
| Trait Hope Scale (THS)(Snyder et al., 1991) | Trait or dispositional | Snyderian cognitive | 12 (4 filler items) | - *I energetically pursue my goals* (self-agency subscale) - *Even when others get discouraged, I know I can find a way to solve the problem* (pathways subscale) | 3 |
| State Hope Scale (SHS)(Snyder et al., 1996) | State or momentary | Snyderian cognitive | 8 | - *At this time, I am meeting the goals I set for myself* (self-agency subscale) - *I can think of many ways to reach my current goals* (pathways subscale) | 3 |
| Children’s Hope Scale - Peabody Treatment Progress Battery (CHS-PTPB)(Dew-Reeves et al., 2012) | Trait or dispositional (adapted brief version of the CHS) | Snyderian cognitive | 4 | - *When I have a problem I can come up with lots of ways to solve it* - *I think I am doing pretty well* | 1 |
| Herth Hope Scale (HHS)(Herth, 1991) | Trait or dispositional | Herth model(Dufault & Martocchio, 1985; Herth, 1991) of hopefulness as encompassing cognitive-temporal, affective-behavioural, and  affiliative-contextual domains | 30 | - *I believe that good is always possible* - *I know my life has meaning and purpose* | 1 |
| Herth Hope Index (HHI)(Herth, 1992a) | Trait or dispositional (adapted brief version of the HHS) | Herth model(Dufault & Martocchio, 1985; Herth, 1991) | 12 | - *I see a light in a tunnel* - *I have a faith that gives me comfort* | 1 |
| Hope scale(Al-Mabuk et al., 1995) | Trait or dispositional and domain-specific (parental relationship, friendships, achievement and affect) | Not stated | 30 | - *There will be more trust in my relationship with my parents* (parental relationship domain) - *I will be able to follow through on tasks* (achievement domain) | 1 |
| Hopefulness about the future scale(Whitaker et al., 2000) | Trait or dispositional | Not stated | 4 | - *I can do just about anything I really set my mind to do* - *I have great faith in the future* | 1 |

**Appendix D: Figure 3 Risk of bias supplementary figure note**

S1. Are there clear research questions? S2. Do the collected data allow to address the research questions? Qualitative: 1.1. Is the qualitative approach appropriate to answer the research question? 1.2. Are the qualitative data collection methods adequate to address the research question? 1.3. Are the findings adequately derived from the data? 1.4. Is the interpretation of results sufficiently substantiated by data? 1.5. Is there coherence between qualitative data sources, collection, analysis and interpretation? Quantitative randomised controlled trial: 2.1. Is randomization appropriately performed? 2.2. Are the groups comparable at baseline? 2.3. Are there complete outcome data? 2.4. Are outcome assessors blinded to the intervention provided? 2.5 Did the participants adhere to the assigned intervention? Quantitative non-randomised 3.1. Are the participants representative of the target population? 3.2. Are measurements appropriate regarding both the outcome and intervention (or exposure)? 3.3. Are there complete outcome data? 3.4. Are the confounders accounted for in the design and analysis? 3.5. During the study period, is the intervention administered (or exposure occurred) as intended? Quantitative descriptive 4.1. Is the sampling strategy relevant to address the research question? 4.2. Is the sample representative of the target population? 4.3. Are the measurements appropriate? 4.4. Is the risk of nonresponse bias low? 4.5. Is the statistical analysis appropriate to answer the research question? Mixed: 5.1. Is there an adequate rationale for using a mixed methods design to address the research question? 5.2. Are the different components of the study effectively integrated to answer the research question? 5.3. Are the outputs of the integration of qualitative and quantitative components adequately interpreted? 5.4. Are divergences and inconsistencies between quantitative and qualitative results adequately addressed? 5.5. Do the different components of the study adhere to the quality criteria of each tradition of the methods involved?

**Appendix E: Characteristics of specific trialled interventions**

| First author | Date | Type | Content | Session N | Interventionist | Delivery | Mode |
| --- | --- | --- | --- | --- | --- | --- | --- |
| Conklin | 2009 | Goal-skills intervention | Psychoeducation on links between goal accomplishment and mood, identification of goals in different life domains, learning skills for goal setting and goal pursuit, group discussions on goal successes and challenges. | 2 | Not stated | Face-to-face | Group |
| Fowler | 2018 | Social Recovery Therapy (Fowler et al., 2013, 2019) (www.socialrecoverytherapy.co.uk) | CBT-based psychosocial/psychotherapeutic intervention with assertive outreach and multisystemic principles involving social recovery formulation and behavioural work in the community. | 0–37 (M= 16·49, SD 8·39) | Intervention-trained Clinical Psychologists and/or CBT therapists | Face-to-face | Individual |
| Gabrielsen | 2019 | Friluftsterapi wilderness therapy | Focused on experience of oneself in wild places. Integrated individual and group-based therapeutic work with the experience of basic outdoor life and activities designed to build group cohesion and engage participants through ecological, psychological and physiological processes. | Eight single days and two overnight trips of three and six days, completed within 8-10 weeks | Therapists with requisite outdoor skills | Face-to-face | Individual and group |
| Gee | 2018 | Social Recovery Therapy (Fowler et al., 2017, 2019) (www.socialrecoverytherapy.co.uk) | CBT-based psychosocial/psychotherapeutic intervention with assertive outreach and multisystemic principles involving social recovery formulation and behavioural work in the community. | Not stated | Intervention-trained Clinical Psychologists and/or CBT therapists | Face-to-face | Individual |
| Gillig | 2019 | Brave Trails’ camp programme (https://www.bravetrails.org/) | The camp schedule is structured around four types of programming: free-choice programs (counsellor-led activities such as swimming, archery, and hiking), workshops (visitor-led workshops on topics such as self-care, meditation and sexual health), build-on programmes (project-based such as film-making), and a social entrepreneurship course, which involves creating and articulating a “story of self”. | Two weeks’ camping | Camp staff who mainly identify as LGBTQ and who have ‘years’ of experience working with youth | Face-to-face | Group |
| Green | 2007 | Life coaching programme (L. S. Green & Grant, 2006; Spence & Grant, 2005) | Solution-focused cognitive-behavioural life coaching, involving the identification of one school and one personal problem and relevant goal setting, action planning, progress monitoring and evaluation. | 10, over two 13-week school terms | Intervention-trained teacher | Face-to-face | Individual |
| Isa | 2018 | Psychoeducation and CBT | Interactive lectures and group discussions focusing on psychoeducation relating to depression, using the cognitive technique of positive self-talk, the promotion of hopefulness and medication adherence, and activity scheduling. | 4, weekly | Psychiatrist | Face-to-face | Group |
| Leibovich | 2020 | Supportive Expressive psychotherapy (Zilcha-Mano et al., 2018) | Psychoeducation using Core Conflictual Relationship Themes concept to understand wishes, expectations and responses in relationships, therapist use of supportive and expressive techniques and the provision of interpretations. | 16, weekly | Intervention-trained psychologist | Face-to-face | Individual |
| Lin | 2013 | Forgiveness intervention (Enright & Fitzgibbons, 2000) | Intervention structured around the Forgiveness Process Model(Enright & Fitzgibbons, 2000). Sessions 1-4 exploring issues and defences, sessions 5-6 instilling hope and commitment to forgiveness, sessions 7-9 focused on forgiveness and sessions 10-11 on its benefits, and 12 was an individual problem and future plan-focused sessions. | 12, weekly | Not stated | Face-to-face | Group, one individual session |
| Lin | 2014 | Grief-processing-based psychological intervention | Warm-up activities, followed by games and activities designed to facilitate discussion, sharing and supporting each other with grief and bereavement. Each session ended with a hug for each participant. | 6, weekly | Clinical academic and postgraduate student trained in family therapy | Face-to-face | Group |
| Metsӓranta | 2019 | Depis.net (Välimäki et al., 2012) e-diary | E-diary given as homework within Depis.net online intervention, with instruction to describe current thoughts, feelings, and moods weekly and additional suggested personal writing tasks relating to weekly intervention themes of well-being, home and family, adolescents’ rights and responsibilities, adolescent depression, and treatment of  adolescents’ depression. | 6 weeks’ suggested homework use | Not applicable | Online | Individual |
| Ritschel | 2011 | Behavioural activation (Dimidjian et al., 2008) adapted for adolescents | Six general phases of treatment: orientation, engaging in activities, problem solving, setting goals and subgoals, practice, and relapse prevention. | 22 over 18 weeks | Intervention-trained doctoral level psychologists and postgraduate students | Face-to-face | Individual, some parent attendance |
| Ritschel | 2016 | Behavioural activation (Dimidjian et al., 2008) adapted for adolescents (Ritschel et al., 2011) | Six general phases of treatment: orientation, engaging in activities, problem solving, setting goals and subgoals, practice, and relapse prevention. | 22 over 18 weeks | Intervention-trained doctoral level psychologists and postgraduate students | Face-to-face | Individual, some parent attendance |
| Sælid | 2017 | Rational Emotive Behaviour Therapy (Dryden et al., 2010) | Identification of a problem or life adversity focus, agreement on a therapeutic goal, education using intervention model and connections between events, behaviours, and beliefs, challenging beliefs and replacing with rational thinking. | 3 | Intervention-trained therapist | Face-to-face | Individual |
| Shepherd | 2018 | SPARX online CBT-based intervention game (Merry et al., 2012) | Seven modules in 3D environment with psychoeducation, missions and puzzles and using avatar and Māori symbols/images e.g. canoes. | Not applicable | Not applicable | Online | Individual |
| Smith | 2011 | Integrated yoga practice with meditation | Warm-up, stretching, and breathing exercises were accompanied by a meditation based on yamas (restraints) and niyamas (observances) of yogic philosophy. | 14 over 7 weeks | Yoga teacher | Face-to-face | Group |
| Teodorczuka | 2019 | Positive psychology intervention (Feldman & Dreher, 2012; Proctor et al., 2011) | Group discussion on topics such as gratitude, and activities including counting blessings, engaging in acts of kindness, identifying and using character strengths, imagining the best possible self, goal mapping, loving kindness mediation and savouring exercises. | 6, weekly | Psychologist | Face-to-face | Group |
| Walsh | 1997 | Art future image intervention (Walsh, 1993; Walsh & Hardin, 1994) | Discussion of future plans and dreams, paper and pencil future-oriented sentence completion activity, further discussion of future occupations and "life dreams", drawing or collaging large self-caricature poster of “future imagined self” using enlarged photo of own face. | 1 | Intervention-trained psychiatric unit nurses | Face-to-face | Group |

*Notes*: M= mean, SD= standard deviation. Additional sub-studies (Anttila et al., 2015; Midgley et al., 2016) to novel trialled interventions not included as sub-study data collection preceded intervention.

**Appendix F: Qualitative study results**

| First author | Date | Epistemological approach | Analytic approach | Data collection tool/s | Higher order themes |
| --- | --- | --- | --- | --- | --- |
| Anttila | 2014 | Not stated | Inductive Thematic Analysis(Braun & Clarke, 2006) | On first access to the Depis.net programme, adolescents were asked to write down their thoughts about their current life situations and perceived concerns in a short essay. | Themes reflect areas of life in which concerns and hopes emerged:   1. Relationships 2. Daily actions 3. Identity 4. Well-being |
| Binder | 2013 | Hermeneutical-phenomenological(Heidegger, 1996; Laverty, 2003; Jonathan A Smith, 2007; Van Manen, 1997) | Explorative-reflexive Thematic Analysis(Binder et al., 2012; Braun & Clarke, 2006) | Interview schedule structured using Bordin’s therapeutic alliance concept(Bordin, 1994). Questions focused on participants’ experiences of early and current treatment, experiences of their therapists and therapeutic relationship, experiences of disclosure, collaborative goal and task identification, and reflexive exploration of the “ideal” adolescent therapist. | 1. Potentially obscuring contact with the unique personhood of the client 2. Providing hope through establishing trust in the therapist’s competence in understanding the problems they face 3. Finding the balance: Perceived relational authenticity allowing for collaboration during professional procedures 4. Sensing a pressure from the system outside the therapeutic dyad |
| Bury | 2007 | Phenomenological | Interpretive Phenomenological Analysis(Osborn & Smith, 1998; Jonathan A. Smith, 1996) | Semi-structured interview schedule developed according to the biographical-interpretive approach(Hollway & Jefferson, 2000) enabling participants to tell their stories of their experiences of psychotherapy | 1. Seeking help and engagement 2. Beginning therapy 3. The therapeutic process 4. Endings |
| Davidson | 2012 | Not stated | Grounded Theory(Corbin & Strauss, 2008) | Focus group and individual interviews, exploring experiences of therapy, the therapeutic relationship, and disclosure of personal information during therapy. | 1. The experience of being in therapy 2. Forming a connection with the therapist |
| Gabrielsen | 2019 | Critical realist | Initial description and analytic resolution using a critical realist model(Danermark et al., 2002) | Fieldwork (participant observation) and two rounds of individual semi-structured interviews. | 1. Influential processes at the time of post-test 2. Post-treatment processing and perceived effects of intervention efforts themselves |
| Gee | 2016 | Critical realist | Inductive Thematic Analysis(Braun & Clarke, 2006) | Semi-structured interviews focused on history of psychological difficulties, experiences accessing services and of trial participation, views on the intervention received, and perceived outcomes. | 1. "*It's just the speaking to someone*": the value of talking 2. "*Just do it*": the importance of activity 3. Motivation to change   Intervention arm only:   1. "*She understood me on a personal level*": the therapeutic relationship 2. Flexibility 3. "*It's given me the tools*": the CBT toolkit 4. No pain, no gain: social recovery therapy as difficult   Control arm only:   1. Allocation ambivalence 2. No treatment, as usual 3. "*I was the one who had to do everything to help overcome it*” |
| Hambridge | 2017 | Pragmatic(Glasgow et al., 1999; Johnson & Onwuegbuzie, 2004) | Inductive and deductive Interpretive Phenomenological Analysis (J. A. Smith et al., 2012) | Individual semi-structured interviews, observations of care farm sessions and secondary document analysis. Interview schedule included motivations for attending, experiences, and perceived benefits, and enjoyed aspects of the care farm, changes to self, life, and relationships since attending and future aspirations. Secondary documents pertained to participants’ histories. | 1. Green environmental engagement 2. Personal functioning 3. Social functioning 4. Personal development 5. Mental health development and behavioural regulation difficulties |
| Leavey | 2005 | Not stated | Grounded Theory | Semi-structured interviews. Post-interview, participants were invited to a feedback and member validation focus group. | 1. Emergence 2. Loss 3. Adaptation 4. Recovery |
| Leibovich | 2020 | Not stated | Illustrative | Psychotherapy recordings | Qualitative data used to illustrate the coding of growth-facilitating interpretations |
| Metsӓranta | 2019 | Not stated | Content analysis(Graneheim & Lundman, 2004) | Participant e-diary entries | 1. Mental disorder 2. Relationship 3. Identity |
| Midgley | 2016 | Not stated | Framework Analysis(Ritchie et al., 2002) | Semi-structured interviews, using Expectations of Therapy Interview(Midgley et al., 2011) | 1. “*I dunno*”: The difficulty of imagining what will happen in therapy 2. The “*talking cure*” 3. “*They talk to you and give you a bit of medicine*”: The therapist as doctor 4. “*Slowly, so slowly I can get there*”: Therapy as a relationship 5. *Just get all this pain over and done with*”: Regaining the old self, or developing new capacities |
| Pingitore | 2017 | Not stated | Qualitative manifest content analysis(Elo & Kyngäs, 2008; Sandelowski, 2000) | Semi-structured, open-ended interviews | 1. Experience of group therapy as a process 2. Benefits of group participation 3. Recommendations for future group psychotherapists 4. Therapeutic factors |
| Rayner | 2018 | Not stated | Inductive Thematic Analysis(Braun & Clarke, 2006) | Semi-structured interview schedule(Simonds et al., 2014) | 1. Ecological systems 2. Youth recovery processes |
| Shepherd | 2018 | Critical realist using Kaupapa Māori research methodology(Health Research Council of New Zealand, 2010) | Inductive Thematic Analysis(Braun & Clarke, 2006) | Semi-structured interviews, involving sharing prayers and food | 1. SPARX is helpful as it taught CBT skills 2. Maori designs assisted with the engagement of Maori adolescents 3. Characters in SPARX provided hope and helpful advice 4. SPARX game design was enjoyable and provided challenging factors 5. SPARX booklet was useful to record participant’s thoughts and feelings |
| Walsh | 1997 | Phenomenological | Phenomenological analysis(Parse, 1993) | Semi-structured interviews, 24-48 hours after intervention and two months following inpatient discharge | Synthesised definition of intervention experience: "An irritating but humorous identity search which rekindles dreams as control over one's future is anticipated." Identified from six themes:   1. Compliant irritation 2. Identity searching 3. Humour reappearing 4. Rekindling dreams 5. Regaining control 6. Pleasant anticipation |
| Watsford | 2013 | Not stated | Inductive Thematic Analysis (Braun & Clarke, 2006) | Semi-structured interviews | 1. Client role expectations 2. Expectations of therapist's role 3. Therapy processes expectations 4. Outcome expectations |
| Weitkamp | 2017 | Not stated | Interpretive Phenomenological Analysis (J. A. Smith et al., 1997; Jonathan A Smith & Osborn, 2008) | Semi-structured interview, using Expectations of Therapy Interview(Midgley et al., 2011) | 1. “*I don’t have a clear image of it, I just believe, if it helps me, it’s good*”—Not knowing but being cautiously hopeful 2. “*This won’t be easy of course, but it is the right way, so to speak*”—Therapy as a long and difficult process 3. "*To be more reflective, well, that I can react differently in the future*”—Therapy as a place to understand oneself and to develop 4. “*That I will find the perfect therapist for me*”—The importance of the professional and interpersonal skills of the therapist |

*Note:* Italics denote verbatim quotation from study participant.

**Appendix G: Barriers to experiencing hopefulness identified by young lived experience experts**

| Demography and lifestyle conditions | - Older age, Physical illness and disability - Financial or time poverty - Lack of independence from family |
| --- | --- |
| Health | - Mixed anxiety and depression, Severe or long-lasting depression, Crisis or inpatient admission |
| Environment | - Rural location, Homelessness - Poor education or illiteracy - Physical or sexual violence or abuse, Systemic racism, Witnessing police brutality, Living within an oppressive regime |
| Mental health services | - Aging out of the range served by youth mental health services - Poor prior experiences of services - Concerns about service confidentiality, especially for young people aged under 18 years - Feeling unable to be honest with a professional due to lack of trust - Feeling that there is a lack of an authentic relationship with professionals - Feeling invalidated or misunderstood by mental health professionals - Feeling that youth mental health problems are not taken seriously - Stigma associated with taking psychiatric medication |
